# Surface‐Associated Proteins on Extracellular Vesicles Remodel the Tumor Microenvironment by Potentiating TGF‐β Signaling in a Contact‐Dependent Manner

**DOI:** 10.1002/advs.202513286

**Published:** 2025-11-30

**Authors:** Chao Li, Agustin Enciso‐Martinez, Lizhe Zhu, Sarah A. Rotman, Peter A. van Veelen, Roman I. Koning, Hailiang Mei, Peter ten Dijke

**Affiliations:** ^1^ Oncode Institute and Department of Cell and Chemical Biology Leiden University Medical Center Leiden 2333 ZC the Netherlands; ^2^ Amsterdam UMC University of Amsterdam Biomedical Engineering & Physics Amsterdam Cardiovascular Sciences Cancer Center Amsterdam Meibergdreef 9 Amsterdam 1105 AZ the Netherlands; ^3^ Amsterdam UMC University of Amsterdam Laboratory of Experimental Clinical Chemistry Laboratory Specialized Diagnostics & Research Department of Laboratory Medicine Meibergdreef 9 Amsterdam 1105 AZ the Netherlands; ^4^ Center for Proteomics and Metabolomics Leiden University Medical Center Leiden 2333 BA the Netherlands; ^5^ Electron Microscopy Facility Department of Cell and Chemical Biology Leiden University Medical Center Leiden 2333 ZC the Netherlands; ^6^ Department of Biomedical Data Sciences Sequencing Analysis Support Core Leiden University Medical Center Leiden 2333 ZC the Netherlands

**Keywords:** cancer‐associated fibroblasts, CD8^+^ T cells, EV corona, EV surface proteins, extracellular vesicles, TGF‐β, TSG6

## Abstract

Cancer‐associated fibroblasts (CAFs) are a major stromal cell type within the tumor microenvironment (TME), where they drive extracellular matrix remodeling and influence tumor progression through the secretion of bioactive molecules. Transforming growth factor‐β (TGF‐β) is a key regulator of CAF activation, yet its impact on the composition and function of extracellular vesicles (EVs) secreted by CAFs remains largely unexplored. Here, it is shown that TGF‐β activation alters the protein cargo and function of CAF‐derived EVs (TGF‐β‐EVs), leading to a distinct enrichment of surface‐associated proteins. One such protein is tumor necrosis factor‐stimulated gene‐6 protein (TSG6), which interacts with receptor CD44 and its ligand hyaluronan on EVs. The EV surface‐associated proteins facilitate EV docking to cell membranes by binding to transmembrane receptors. Elevated TSG6 on CAF‐derived EV surface promotes the clustering of the co‐receptor CD44 and TGF‐β type I receptor (TGFBR1) on recipient cells, enhancing TGF‐β signaling. Functionally, TGF‐β‐EVs further activate CAFs and contribute to CD8^+^ T cell immunosuppression, thereby promoting cancer progression. Overall, the findings reveal a contact‐dependent mechanism by which CAF‐derived EVs influence cellular signaling in the TME, suggesting a broader paradigm in which EV surface‐associated proteins regulate receptor clustering and downstream signaling, particularly in cells with low EV uptake.

## Graphical abstract

Extracellular vesicles (EVs) released from TGF‐β‐activated CAFs are enriched with ECM proteins such as TSG6 and THBS1, which facilitate their binding to recipient cell membranes. This EV–cell interaction promotes the clustering of CD44 and TGF‐β receptors on the target cell surface, thereby potentiating TGF‐β signaling activity. This study highlights a novel paradigm in EV‐mediated intercellular communication.

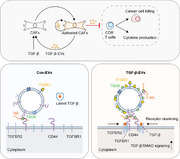

## 1 Introduction

Cancer‐associated fibroblasts (CAFs) are a major component of the tumor stroma within the tumor microenvironment (TME) across multiple cancer types.^[^ ^1^ ^]^ CAFs are heterogeneous in both origin and function, and they actively contribute to cancer progression through complex interactions with other cell types within the TME.^[^ ^2^, ^3^ ^]^ CAFs are highly plastic, capable of sensing external stimuli and responding by activating signaling cascades that lead to their transition into an activated state. A key cytokine frequently present at high levels in the TME is transforming growth factor‐beta (TGF‐β). TGF‐β ligands interact with TGF‐β type II receptors (TGFBR2), which recruit and phosphorylate TGF‐β type I receptors (TGFBR1, also known as activin receptor‐like kinase 5, ALK5). Subsequently, either SMAD or non‐SMAD signaling pathways can be activated.^[^ ^4^ ^]^ TGF‐β contributes to immune suppression within the TME, affecting T cells, natural killer cells, and other immune cells, thereby promoting tumor immune evasion and poor responses to cancer immunotherapy.^[^ ^5^ ^]^ TGF‐β is also a potent inducer of fibroblast activation into CAFs.^[^ ^6^ ^]^ Activated CAFs produce and remodel the extracellular matrix (ECM), and release soluble factors and extracellular vesicles (EVs) to communicate with other cell types in the TME.^[^ ^6^ ^]^

EVs are membrane‐enclosed particles released by cells, mediating intercellular communication through cargo transfer or membrane contact.^[^ ^7^ ^]^ Given the abundance of CAFs in the TME across multiple cancer types, especially in pancreatic and breast cancer, CAF‐derived EVs play a critical role in cell‐to‐cell communication and TME remodeling.^[^ ^8^ ^]^ CAF‐derived EVs have been shown to promote cancer cell proliferation, migration, and therapeutic resistance, while also inducing metabolic reprogramming in cancer cells by delivering functional cargo.^[^ ^9^ ^]^ Through these actions, they contribute to cancer development. Although CAF‐derived EVs have been implicated in immune evasion by inducing programmed death‐ligand 1 (PD‐L1) expression in cancer cells through various mechanisms,^[^ ^10^ ^]^ the direct effects of CAF‐derived EVs on immune cell functions remain largely unexplored.

Historically, EV functional studies have primarily focused on the molecular cargo contained within EVs, including proteins and nucleic acids. However, EVs have a relatively large surface area compared to their volume due to their small size.^[^ ^11^ ^]^ The EV surface is a versatile platform for presenting proteins, nucleic acids, and extracellular matrix components.^[^ ^12^ ^]^ EV surface molecules can be derived from parental cells and be acquired from the extracellular environment. This coronal layer surrounding the EV membrane creates a highly interactive and dynamic area that facilitates intercellular communication and affects EV properties including function, uptake, and in vivo distribution.^[^ ^12^, ^13^, ^14^ ^]^

Many signaling pathways are initiated through ligand‐induced dimerization or multimerization of cell surface receptors. The clustering or higher‐order oligomerization of receptor complexes can further amplify and sustain downstream signaling, including epidermal growth factor receptor (EGFR) receptors,^[^ ^15^ ^]^ tumor necrosis factor (TNF) receptors,^[^ ^16^ ^]^ and Fas receptors.^[^ ^17^ ^]^ The receptor aggregation of Fas receptors is essential for effective apoptotic signaling.^[^ ^17^, ^18^ ^]^ Notably, the Fas ligand displayed on the surface of synthetic vesicles can significantly induce Fas signaling in target cells and subsequent apoptosis, compared to the soluble Fas ligand, by promoting the sequestration and multimerization of the Fas receptor.^[^ ^19^ ^]^ However, whether the surface‐associated proteins on cell‐derived EVs can interact with target cell receptors to trigger cell signaling and whether this mechanism applies to other signaling pathways remains unknown.^[^ ^12^, ^20^, ^21^ ^]^

In this study, we revealed that TGF‐β can significantly upregulate the amount of surface‐associated proteins on CAF‐derived EVs. We identified multiple upregulated ECM proteins, including tumor necrosis factor‐stimulated gene‐6 protein (TSG6) and thrombospondin 1 (THBS1), which were shown to be essential for EV‐mediated TGF‐β signaling. EVs derived from TGF‐β‐activated CAFs promote the clustering of co‐receptor CD44 and TGF‐β receptors on the target cell surface, thereby enhancing the cellular responsiveness to TGF‐β ligands. Functionally, EVs from TGF‐β‐activated CAFs can further enhance the activation of neighboring CAFs and contribute to the immunosuppression of CD8^+^ T cells, ultimately facilitating cancer progression.

## 2 Results

### 2.1 CAF Activation by TGF‐β and Subsequent EV Isolation

**Figure 1:**
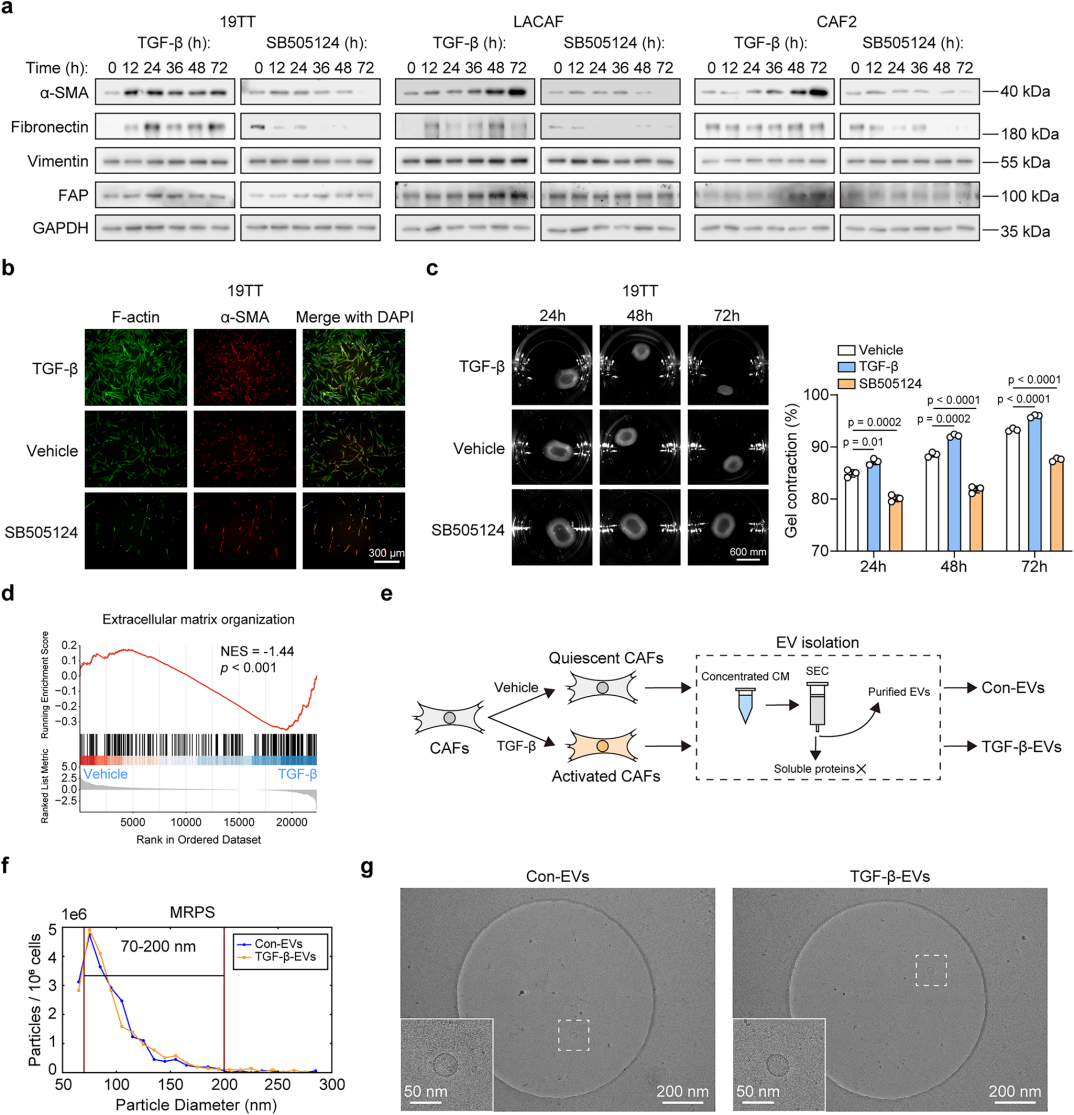
CAF activation by TGF‐β and subsequent EV isolation. a) Western blot analysis of the expression levels of α‐SMA, fibronectin, Vimentin, and FAP in response to treatment with TGF‐β (2.5 ng mL^−1^) or a selective small molecule TGFBR1 inhibitor SB505124 (1 µm) in three immortalized breast CAF lines: 19TT, LACAF and CAF2. GAPDH, loading control. Representative of *n* = 3 experiments. Ultra‐sensitive enhanced chemiluminescent (ECL) substrate was used to detect fibronectin in SB505124‐treated cells. b) Phalloidin (to selectively label filamentous actin) and α‐SMA fluorescent staining in 19TT cells to assess the effect of TGF‐β and SB505124 on CAF activation. CAFs were incubated with treatments for 2 days. The cell nuclei were stained with DAPI. The scale bar represents 300 µm. Representative of *n* = 3 experiments. c) Collagen gel contraction assay using 19TT cells to assess the effects of TGF‐β and SB505124 on CAF activation. Representative images were shown in the left panel, and the scale bar represents 600 mm. The percentage of collagen gel contraction was quantified in the right panel. Means ± SD, *n* = 3 biological replicates, one‐way ANOVA with Dunnett's test. Representative of *n* = 3 experiments. d) Gene set enrichment analysis (GSEA) of extracellular matrix organization pathway using RNA‐Seq transcriptional profiling data from 19TT CAFs treated with vehicle control and TGF‐β (2.5 ng mL^−1^) for 2 days. e) Schematic of the EV isolation workflow. f) Particle concentrations and size distributions of con‐EVs and TGF‐β‐EVs derived from 19TT cells characterized by microfluidic resistive pulse sensing (MRPS). Representative of *n* = 3 experiments. g) Representative cryo‐EM images of con‐EVs and TGF‐β‐EVs, from >200 and >500 images, respectively. EVs were derived from 19TT cells.

In tumor development, TGF‐β activates normal fibroblasts into CAFs,^[^ ^6^ ^]^ and serves as a primary driver of their differentiation into myofibroblastic CAFs (myCAFs).^[^ ^22^ ^]^ To explore whether CAFs undergo further activation in response to TGF‐β, we selected three immortalized breast CAF cell lines for investigation. First, we examined their responsiveness to TGF‐β by measuring the mRNA level of *SERPINE1*, which encodes plasminogen activator inhibitor 1 (PAI1), a prototypic TGF‐β target gene driven by SMAD proteins for transcriptional activation. A significant increase in *SERPINE1* mRNA expression was observed following TGF‐β treatment (Figure S1a–c, Supporting Information). Furthermore, the mRNA levels of *ACTA2*, which encodes α‐smooth muscle actin (α‐SMA), and fibroblast activating protein (*FAP*), both CAF activation markers, were significantly upregulated by TGF‐β after 36 h in all three CAF lines (Figure S1a–c, Supporting Information). In addition, a SMAD3‐driven transcriptional (CAGA)_12_‐enhanced green fluorescent protein (EGFP) reporter assay was performed to examine the responsiveness to TGF‐β in 19TT CAFs, and an increase in EGFP signal was observed following TGF‐β treatment (Figure S1d, Supporting Information). Next, we treated three CAF lines with TGF‐β or a selective small molecule TGFBR1 inhibitor SB505124 for 0 to 72 h and analyzed the protein levels of multiple CAF activation markers, as these markers may exhibit varying responses due to the heterogeneity of CAFs.^[^ ^2^ ^]^ After treatment with TGF‐β, the expression of α‐SMA increased in all three CAF lines, while treatment with SB505124 decreased expression (**Figure** **1**a). In the 19TT CAF line, fibronectin expression increased following TGF‐β treatment and decreased with SB505124 treatment. In both LACAF and CAF2 cells, SB505124 induced a declining trend in fibronectin levels. By contrast, fibronectin levels fluctuated in TGF‐β‐treated LACAF cells and remained relatively stable in TGF‐β‐treated CAF2 cells. The level of Vimentin showed a marginal increase in all three CAF lines after TGF‐β treatment, with a decrease only noted in LACAF following treatment with SB505124. The expression of FAP increased in response to TGF‐β in LACAF and CAF2 cells (Figure 1a). Overall, these three CAF cell lines showed a similar activation response to TGF‐β.

For further functional validation of CAF activation by TGF‐β, we used 19TT CAF cells. Immunofluorescence staining showed that the filamentous (F‐) actin stress fibers were more prominent and organized in parallel bundles in TGF‐β‐treated CAFs than control CAFs, accompanied by higher expression and incorporation of α‐SMA protein into the stress fibers. This effect was inhibited by treatment with SB505124 (Figure 1b). A hallmark of activated myofibroblasts or myCAFs is their acquisition of contractile properties characteristic of smooth muscle cells. To examine these contractile properties, a collagen gel contraction assay was performed with CAFs. After TGF‐β treatment, increased contraction was observed in CAF‐populated collagen gels compared to the control group. In contrast, SB505124 treatment inhibited this contraction (Figure 1c). Additionally, gene set enrichment analysis (GSEA) was conducted using RNA‐Seq transcriptional profiling data, revealing that TGF‐β significantly upregulated the ECM organization signature of 19TT CAFs. We also investigated the expression of commonly used markers to identify different CAF subtypes, including myCAFs, inflammatory CAFs (iCAFs), and antigen‐presenting CAFs (apCAFs).^[^ ^1^, ^2^ ^]^ The myCAF markers were highly expressed in both control and TGF‐β‐treated 19TT CAFs. In contrast, mRNA expressions of iCAF and apCAF markers were extremely low, further suggesting the classification of 19TT CAFs as myCAFs (Figure S1e, Supporting Information). These results indicate that TGF‐β can induce CAF activation in 19TT cells.

Next, we isolated EVs from the conditioned medium of control and TGF‐β‐activated CAFs using size exclusion chromatography (SEC), a gentle method for EV isolation that isolates EVs while removing soluble proteins (Figure 1e). The medium supplemented with EV‐depleted serum was utilized for EV preparation. The stimulatory effect of TGF‐β on CAF activation in cultures with EV‐depleted serum was validated (Figure S2a, Supporting Information). The isolation of 19TT‐ and LACAF‐derived EVs was confirmed by the detection of the tetraspanin markers CD9, CD63 and CD81, which are enriched in EV membranes (Figure S2b, Supporting Information). EV samples were further characterized using microfluidic resistive pulse sensing (MRPS, Figure 1f), cryo‐electron microscopy (cryo‐EM, Figure 1g) and nanoparticle tracking analysis (NTA, Figure S2c,d, Supporting Information) to evaluate size distribution, particle concentration, integrity, and morphology. MRPS, which has a lower detection limit than NTA, allowed for the detection of smaller EVs (>70 nm). EV particle numbers were normalized to the number of EV‐releasing cells. We found that TGF‐β treatment did not affect EV release in 19TT cells (Figure 1f). Cryo‐EM analysis, which enables the detection of EVs across all size ranges, revealed that TGF‐β decreased the median particle size of EVs (Figure S2e, Supporting Information). Analysis of more than 200 cryo‐EM images per EV sample indicated high sample purity, with no visible protein or EV aggregates (Figure 1g; Figure S2f, Supporting Information).

### 2.2 EVs Derived from TGF‐β‐Activated CAFs Promote CAF Activation and Immune Suppression

We further assessed the function of CAF derived‐EVs on various cell types in the TME by treating target cells an equal number of EVs (4 × 10^8^ particles mL^−1^ as measured by NTA), which are roughly from an equal number of EV‐releasing cells (Figure 1f). We first tested 19TT‐ and LACAF‐derived EVs on breast cancer cell lines, specifically triple‐negative MDA‐MB‐231 cells and luminal MCF‐7 cells. The proliferation of MDA‐MB‐231 and MCF‐7 cells was not affected by the treatment of con‐EVs and TGF‐β‐EVs from 19TT and LACAF CAFs (Figure S3a–d, Supporting Information). We next tested the effects of con‐EVs and TGF‐β‐EVs on MDA‐MB‐231 and MCF‐7 cell migration using a wound healing assay, but no significant effect was observed (Figure S3e–h, Supporting Information). The uptake of 19TT CAF‐derived EVs by MDA‐MB‐231 and MCF‐7 cells was assessed using the lipophilic green fluorescent dye PKH67 to label the EVs. Fluorescence analysis confirmed significant EV internalization in both cell lines (Figure S3i, Supporting Information). These data suggest that while both con‐EVs and TGF‐β‐EVs were internalized by breast cancer cells, they did not significantly impact cell proliferation or migration.

**Figure 2:**
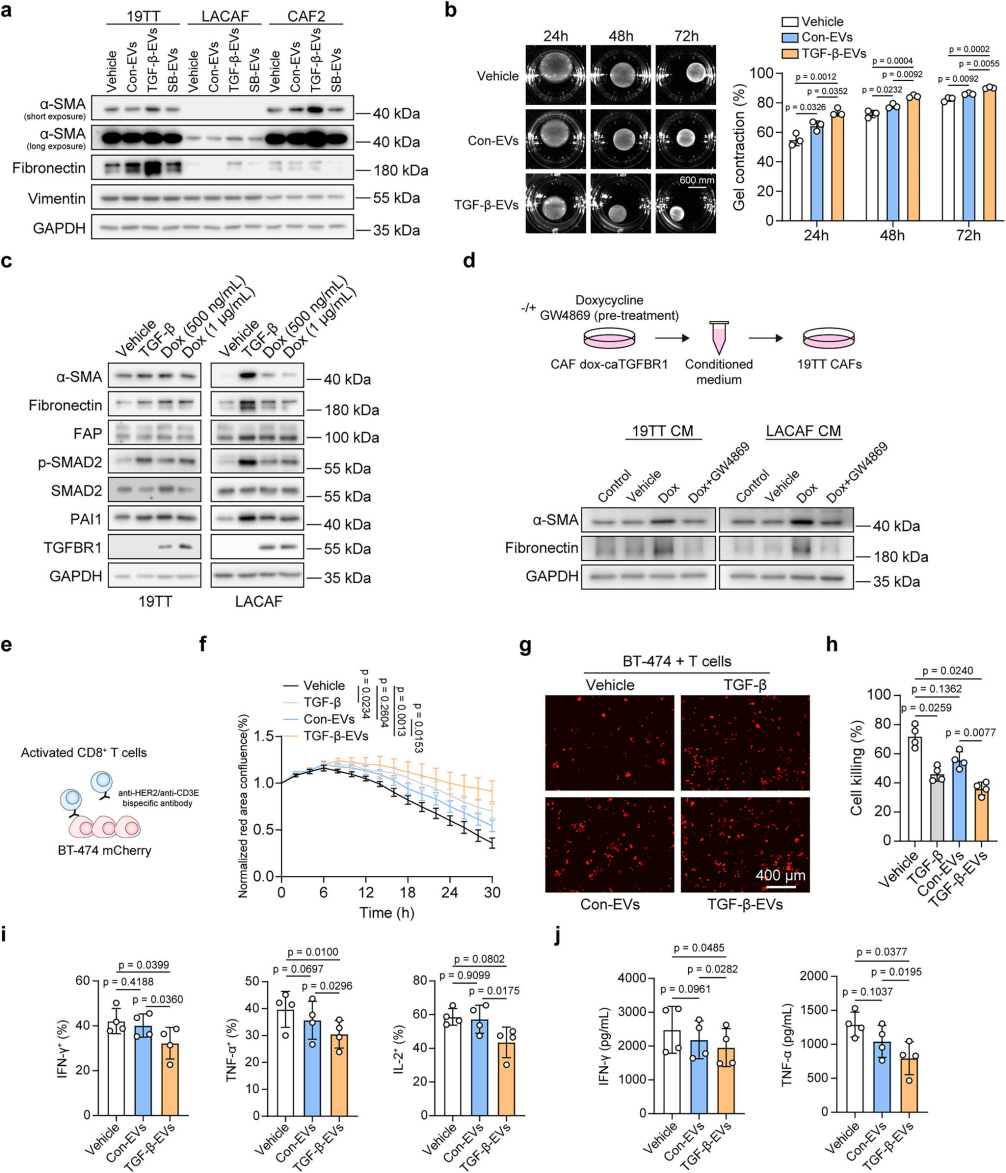
The effect of CAF‐derived EVs on CAFs and CD8^+^ T cells. a) Western blot analysis showing the effect of different EVs on the activation of 19TT, LACAF, and CAF2 cells. Con‐EVs, TGF‐β‐EVs, and SB‐EVs (derived from SB505124‐treated cells) were derived from 19TT cells. CAFs were treated for 2 days. GAPDH, loading control. Representative of *n* = 3 experiments. b) Collagen gel contraction assay using 19TT cells to assess the effect of con‐EVs and TGF‐β‐EVs on CAF activation. Representative images were shown in the left panel, and the scale bar represents 600 mm. The quantification of the percentage of collagen gel contraction was shown in the right panel. Means ± SD, *n* = 3 biological replicates, one‐way ANOVA with Turkey's test. Representative of *n* = 3 experiments. c) Validation of the expression of TGFBRI in doxycycline (dox)‐induced constitutively kinase‐active TGFBR1 (ca‐TGFBR1, T204D) stable expressed 19TT and LACAF cells, and the effect of induced expression of ca‐TGFBR1 on TGF‐β/SMAD signaling and CAF activation. CAFs were treated for 2 days. GAPDH, loading control. Representative of *n* = 3 experiments. d) Schematic of conditioned medium (CM) preparation from dox‐caTGFBR1 stable expressed 19TT and LACAF cells, with or without Dox (500 ng mL^−1^) treatment and GW4869 (5 µm) pre‐treatment. 19TT CAFs were incubated with conditioned medium for 2 days to assess their activation states. GAPDH, loading control. Representative of *n* = 3 experiments. e) Schematic of the co‐culture killing assay involving activated human primary CD8^+^ T cells and human BT‐474 mCherry breast cancer cells, mediated by anti‐HER2/anti‐CD3E bispecific antibody (100 ng mL^−1^). BT‐474 mCherry cells were seeded at a density of 5000 cells per well, and activated CD8⁺ T cells were added at an effector‐to‐target (E:T) ratio of 4:1. f,g) Real‐time tracking of BT‐474 mCherry cells co‐cultured with activated CD8^+^ T cells with indicated treatments, and representative images of different conditions are shown. The scale bar represents 400 µm. Means ± SD, *n* = 3 biological replicates, one‐way ANOVA with Tukey's test. Representative of *n* = 4 donors. h) Quantification of the cytotoxicity of CD8^+^ T cells in the co‐culture. The percentages of cancer cells killed by CD8^+^ T cells were calculated using the cancer cell monoculture as a control. One‐way repeated measures ANOVA with Tukey's test with *n* = 4 donors. i) Flow cytometry quantification of IFN‐γ, TNF‐α, and IL‐2 expression in activated CD8^+^ T cells treated with EVs or vehicle control for 2 days. One‐way repeated measures ANOVA with Tukey's test with *n* = 4 donors. j) Secreted IFN‐γ and TNF‐α from activated CD8^+^ T cells measured by ELISA assay. CD8^+^ T cells were treated with EVs or vehicle control for 2 days. One‐way repeated measures ANOVA with Tukey's test with *n* = 4 donors.

Next, the effects of 19TT CAF‐derived EVs were evaluated on different CAF cell lines. We observed that TGF‐β‐EVs promoted CAF activation by increasing α‐SMA and fibronectin expression (**Figure** **2**a). The effect of TGF‐β‐EVs on CAF activation in 19TT cells was further validated using a collagen gel contraction assay and actin stress fiber staining. TGF‐β‐EV‐treated CAFs showed increased collagen gel contraction and more pronounced actin stress fiber formation, indicating CAF activation (Figure 2b; Figure S4a,b, Supporting Information). CAF proliferation was not affected by EV treatments (Figure S4c, Supporting Information).

To bypass the addition of exogenous TGF‐β to CAFs, we constructed doxycycline (Dox)‐induced constitutively kinase‐active TGFBR1 (ca‐TGFBR1, T204D)^[^ ^23^ ^]^ stable expression cell lines using 19TT and LACAF cells. We validated the ectopic expression of TGFBR1 in these two CAF lines upon treatment with Dox, and the subsequent activation of TGF‐β/SMAD signaling and CAF activation (Figure 2c). Next, we pre‐treated the CAFs with Dox and GW4869, an exosome biogenesis inhibitor,^[^ ^24^ ^]^ for 2 days. The medium was then replaced with fresh medium, with or without Dox, and the cells were incubated for an additional day. The conditioned medium was collected from different conditions and centrifuged to remove potential cells and cell debris, and the resulting conditioned medium was used to treat other CAF cells as an indirect co‐culture assay (Figure 2d). The conditioned medium from activated CAFs by Dox‐induced expression of ca‐TGFBR1 promoted CAF activation (Figure 2d). When CAFs were pre‐treated with GW4869, the conditioned medium from activated CAFs by ca‐TGFBR1 lost its ability to induce CAF activation (Figure 2d). These results suggest that EVs derived from TGF‐β signaling‐activated CAFs can participate in a feedforward loop of CAF activation, and promote the activation of neighboring CAFs.

Within the TME, the cytotoxic CD8^+^ T cells of the adaptive immune system are the most potent effectors in the anticancer immune response and constitute the backbone of cancer immunotherapy.^[^ ^25^ ^]^ Therefore, CD8⁺ T cells were used as a representative immune cell type to assess the effect of CAF‐derived EVs on the cancer immune response. We first examined the effect of EVs on the cancer cell‐killing ability of activated CD8^+^ T cells, using a co‐culture system with fluorescent mCherry protein stable expressing human epidermal growth factor receptor positive (HER2^+^) BT‐474 breast cancer cells and human primary CD8^+^ T cells, whereby CD8^+^ T cell‐cancer cell killing was mediated by anti‐HER2/anti‐CD3E bispecific antibody (Figure 2e). By tracking BT‐474 cells in real‐time, we found that more cancer cells survived during co‐culture in the TGF‐β‐EV treatment group (Figure 2f,g). The cancer‐killing ability of CD8^+^ T cells was significantly inhibited by TGF‐β‐EVs (Figure 2h). CAF‐derived EVs did not affect the proliferation of BT‐474 cells, consistent with our observations for MDA‐MB‐231 and MCF‐7 cells (Figure S4d, Supporting Information). The proliferation of CD8^+^ T cells was also unaffected by CAF‐derived EVs during coculture (Figure S4e, Supporting Information). Moreover, TGF‐β further enhanced the suppressive effect of TGF‐β‐EVs on the cytotoxic activity of CD8^+^ T cells (Figure S4f,g, Supporting Information). In addition, TGF‐β‐EVs suppressed the production of interferon‐gamma (IFN‐γ), tumor necrosis factor‐alpha (TNF‐α) and interleukin‐2 (IL‐2) from activated CD8^+^ T cells, compared to the con‐EVs and control treatment groups (Figure 2i,j; Figure S4h, Supporting Information).

To explore whether the effect of the EVs derived from TGF‐β‐activated CAFs on the immunosuppression of CD8^+^ T cells also applies to the mouse immune cells, we isolated EVs from primary mammary fibroblasts from BALB/c mice. We activated the primary fibroblast using TGF‐β to mimic the activated CAFs, and we confirmed the fibroblast activation by detecting the CAF‐associated protein markers and stress fiber staining (Figure S4i,j, Supporting Information). Mouse ovalbumin (OVA)‐specific, major histocompatibility complex (MHC) class I‐restricted (OT‐I) CD8^+^ T cells and mouse KPC3 pancreatic cancer cells with stably expressed GFP and OVA were used for the co‐culture killing assay. The OT‐I CD8^+^ T cells can specifically recognize the MHC class I presented OVA antigen on KPC3 cells.^[^ ^26^ ^]^ Using this system, we found that EVs derived from TGF‐β‐activated fibroblasts inhibited the cancer cell‐killing ability of OT‐I CD8^+^ T cells, consistent with the results from human cells (Figure S4k, Supporting Information).

Taken together, the EVs derived from TGF‐β‐activated CAFs can promote CAF activation, and induce immunosuppression of CD8^+^ T cells.

### 2.3 Identification of Multiple Proteins in TGF‐β‐EVs that are Essential for CAF‐Derived EV Function on Target Cells

**Figure 3:**
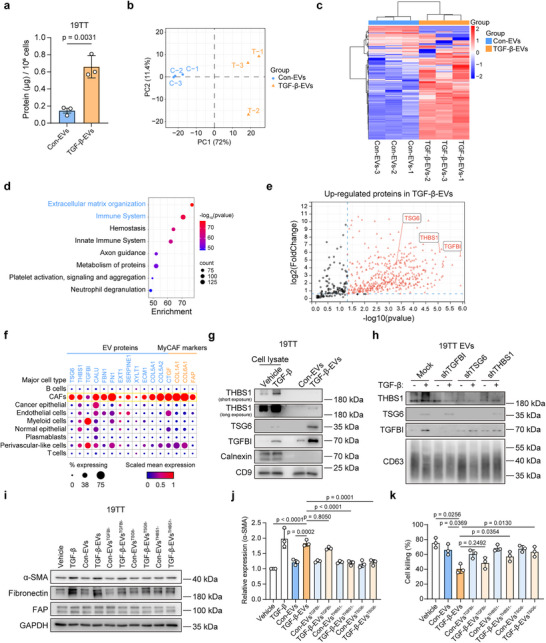
Analysis of top upregulated proteins in TGF‐β‐EVs identified by quantitative proteomics. a) Protein quantification of EVs from vehicle control versus TGF‐β‐treated 19TT cells. EVs were normalized to an equal number of EV releasing cells. Means ± SD, *n* = 3 biological replicates, unpaired student's *t*‐test. Representative of *n* = 3 experiments. b) Principal component analysis (PCA) of proteomic results. c) Heatmap showing sample clustering and expression levels of proteins identified from proteomic analysis. The expression values of proteins are *z*‐score transformed. d) Reactome pathway analysis of the upregulated proteins in TGF‐β‐EVs compared to con‐EVs. Fisher's exact test with false discovery rate (FDR) correction. The presented pathways are among the top 8 enriched pathways. e) Volcano plots showing upregulated proteins in TGF‐β‐EVs compared to con‐EVs. f) Scaled mRNA expression of corresponding genes for several top upregulated proteins in TGF‐β‐EVs across different breast cancer cell types, based on a single‐cell RNA sequencing dataset (GSE176078). g) Western blot analysis of THBS1, TSG6, and TGFBI expression in 19TT cells treated with vehicle control or TGF‐β, and con‐EVs and TGF‐β‐EVs derived from 19TT cells. Representative of *n* = 3 experiments. h) Western blot analysis of THBS1, TSG6, and TGFBI expression levels in con‐EVs and TGF‐β‐EVs, which were derived from control cells, TGFBI‐depleted cells, TSG6‐depleted cells, and THBS1‐depleted cells respectively. Representative of *n* = 3 experiments. i) Western blot analysis of α‐SMA, fibronectin, and FAP expression levels in response to indicated treatments using 19TT cells. CAFs were treated for 2 days. GAPDH, loading control. Representative of *n* = 3 experiments. j) Quantification of relative protein expression of α‐SMA from (i). Means ± SD, *n* = 3 independent experiments, one‐way ANOVA with Tukey's test. k) Quantification of CD8^+^ T cell cytotoxicity in co‐culture with BT‐474 mCherry cancer cells under the indicated treatments. One‐way repeated measures ANOVA with Tukey's test with *n* = 3 donors.

To investigate how the altered function of TGF‐β‐EVs relates to changes in EV proteins, we normalized the total EV protein amount to the number of EV‐releasing cells and EV particle counts measured by MRPS, and analyzed the EV proteomic content. The protein amount was significantly upregulated in TGF‐β‐EVs using both normalization methods (**Figure** **3**a; Figure S5a, Supporting Information). We performed label‐free proteomics utilizing data‐independent analysis (DIA) with an equal number of EVs measured by MRPS (Appendix S1, Supporting Information). Principal component analysis (PCA) revealed a high degree of similarity in the proteomic content of EVs among biological replicates, while clearly distinguishing between con‐EV and TGF‐β‐EV samples (Figure 3b). Compared to the con‐EV group, the TGF‐β‐EV group exhibited a markedly higher number of upregulated proteins than downregulated ones (Figure 3c), aligning with the significantly increased total protein amount in TGF‐β‐EVs (Figure 3a). We conducted Reactome pathway analysis^[^ ^27^ ^]^ for the upregulated proteins in TGF‐β‐EVs, revealing that the ECM organization and immune system pathways were among the highest ranked (Figure 3d). This enrichment of immune system pathways suggests potential alterations in immune regulation, which may be associated with the suppressed CD8⁺ T‐cell function observed in our experiments.

Further investigation of the upregulated proteins revealed many ECM proteins, consistent with the enriched ECM organization pathway (Figure 3d,e). We selected several top upregulated proteins in TGF‐β‐EVs and analyzed the mRNA expression of their corresponding genes in different cell types from breast cancers using a single‐cell RNA sequencing dataset (Figure S5b, Supporting Information).^[^ ^28^ ^]^ We discovered that most of these genes exhibited the highest mRNA expression in CAFs compared with other cell types, suggesting their unique CAF origin in the TME (Figure 3f). Of note, TGF‐β3, the recombinant TGF‐β ligand used for cell treatments, was not detected among the EV proteins. Although seven peptides corresponding to latent TGF‐β1 (L‐TGF‐β1) were identified, none matched the amino acid sequence of TGF‐β3, suggesting that the TGF‐β‐EVs were not contaminated with exogenous TGF‐β3 (Figure S5c, Supporting Information).

We selected several TGF‐β‐EV proteins including THBS1, TSG6, and TGF‐β‐induced protein (TGFBI), for analysis of their potential roles in TGF‐β‐EV function. The selection was based on their strong upregulation in TGF‐β‐EVs and relatively high basal expression, the latter making them easier to detect and manipulate (Figure 3e). The mRNA expression of their corresponding genes was also elevated by TGF‐β treatment in 19TT CAFs (Figure S5d, Supporting Information). We first validated the upregulation of THBS1, TSG6, and TGFBI in TGF‐β‐EVs, and observed that their protein expression increased in TGF‐β‐activated CAFs (Figure 3g). Next, knockdown cell lines for THBS1, TSG6, and TGFBI were constructed using shRNAs (Figure S5e, Supporting Information). The depletion of these proteins in EVs from their corresponding knockdown cell lines was confirmed (Figure 3h). In 19TT CAFs, TGFBI depletion led to a downregulation of THBS1 expression (Figure S5f, Supporting Information), which may have contributed to a reduced THBS1 level in EVs (Figure 3h). We then examined the effects of con‐EVs and TGF‐β‐EVs, with protein depletion, on CAF activation. In the TGF‐β‐EV^TGFBI−^ treatment group, the expression of α‐SMA only showed a slight reduction compared to the TGF‐β‐EV treatment group (Figure 3i,j). In the TGF‐β‐EV^TSG6−^ and TGF‐β‐EV^THBS1−^ treatment groups, the expression of α‐SMA remained almost unchanged compared to con‐EV^TSG6−^, con‐EV^THBS1−^, and con‐EV treatment groups (Figure 3i,j). Additionally, fibronectin expression significantly decreased in TGF‐β‐EV^TSG6−^ and TGF‐β‐EV^THBS1−^ treatment groups compared to TGF‐β‐EV treatment group (Figure 3i), suggesting that TSG6 and THBS1 in EVs play a significant role in CAF activation. The depletion of TSG6 and THBS1 also mitigated the suppressive effect of TGF‐β‐EVs on CD8^+^ T cell‐mediated cancer cell killing and cytokine secretion. In contrast, the effect of TGFBI depletion was not as pronounced as that of TSG6 and THBS1 depletion (Figure 3k; Figure S5g,h, Supporting Information).

To examine how the EV protein composition from TGF‐β‐treated CAFs differed from that of TGF‐β‐treated cancer cells, we compared the upregulated proteins in TGF‐β‐EVs from 19TT CAFs and MDA‐MB‐231 cells, which is part of our previous published proteomics dataset.^[^ ^29^ ^]^ An increased protein level of THBS1 and TGFBI was also observed in TGF‐β‐EVs from MDA‐MB‐231 cells, while the increased protein level of TSG6 and its presence was only detected in TGF‐β‐EVs from CAFs (Figure S5i, Supporting Information). We used MDA‐MB‐231 cell‐derived EVs to treat CAFs. In contrast, TGF‐β‐EVs from MDA‐MB‐231 cells did not promote CAF activation, suggesting that TSG6 may play a unique role in the function of CAF‐derived EVs (Figure S5j, Supporting Information).

### 2.4 TGF‐β‐EVs Induce TGF‐β Signaling in Target Cells

**Figure 4:**
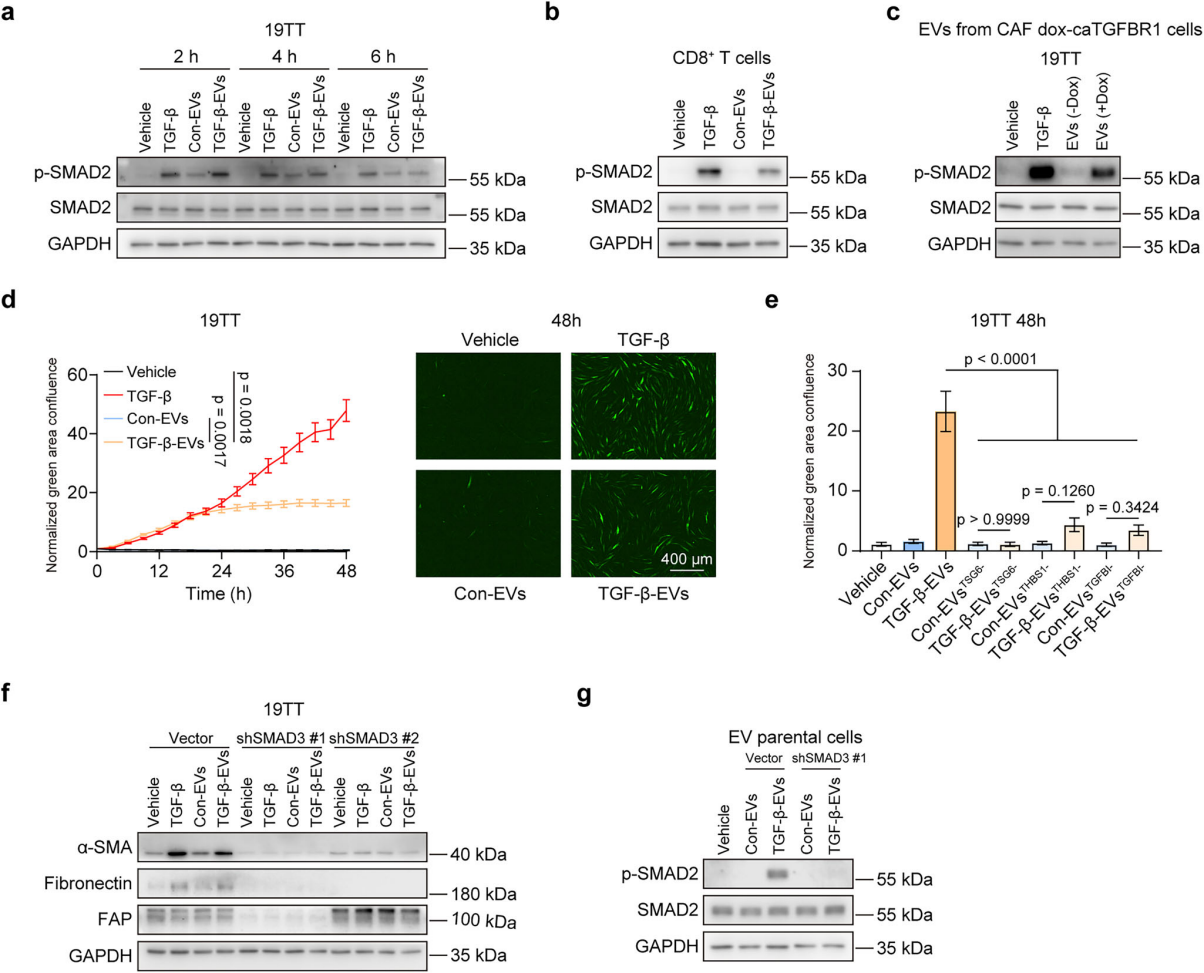
The effect of CAF‐derived EVs on TGF‐β signaling in target cells. a) Western blot analysis of p‐SMAD2 levels in 19TT cells treated with 19TT cell‐derived con‐EVs and TGF‐β‐EVs. GAPDH, loading control. Representative of *n* = 3 experiments. b) Western blot analysis of p‐SMAD2 levels in activated CD8^+^ T cells in response to 19TT cell‐derived con‐EVs and TGF‐β‐EVs. T cells were incubated with indicated treatments for 2 h. GAPDH, loading control. Representative of *n* = 3 experiments. c) Western blot analysis of p‐SMAD2 levels in 19TT cells in response to EVs derived from dox‐caTGFBR1 stable expressed 19TT cells, with or without Dox (500 ng mL^−1^) treatment. 19TT cells were incubated with indicated treatments for 2 h. GAPDH, loading control. Representative of *n* = 3 experiments. d) Real‐time imaging of (CAGA)_12_‐EGFP reporter‐stably‐expressing 19TT CAFs incubated with indicated treatments. The quantification of normalized green area confluence is shown. Means ± SD. Representative images after 48 h of treatment are shown in the right panel. The scale bar represents 400 µm. Representative of *n* = 3 experiments. e) The quantification of normalized green area confluence at 48 h in (CAGA)_12_‐EGFP reporter‐stably‐expressing 19TT CAFs incubated with indicated treatments. Means ± SD. Representative of *n* = 3 experiments. f) Western blot analysis of α‐SMA, fibronectin and FAP expression levels in 19TT WT cells and 19TT SMAD3 depletion cells in response to 19TT cell‐derived con‐EVs and TGF‐β‐EVs. 19TT cells were treated for 2 days. GAPDH, loading control. Representative of *n* = 3 experiments. g) Western blot analysis of the effect of EVs derived from 19TT SMAD3‐depleted cells on TGF‐β signaling. 19TT CAFs were treated with EVs for 2 h. Representative of *n* = 3 experiments.

Since TGF‐β signaling is one of the most potent inducers of CAF activation and T cell immunosuppression in the TME,^[^ ^6^, ^30^ ^]^ we examined whether TGF‐β‐EVs exert their function by regulating TGF‐β signaling in target cells. We found that TGF‐β signaling was indeed activated by TGF‐β‐EVs in CAFs and CD8^+^ T cells, as evidenced by the elevated expression of TGFBR1 effector p‐SMAD2 (**Figure** **4**a,b). Besides TGF‐β3, which was used throughout the study, EVs derived from TGF‐β1‐activated CAFs also showed a consistent ability to activate TGF‐β signaling in CAFs (Figure S6a,b, Supporting Information). In addition, to eliminate the contribution of potential residual exogenous TGF‐β ligand, we isolated EVs from dox‐caTGFBR1 stable expressed 19TT cells, with or without Dox treatment. We found that EVs derived from Dox‐induced TGFBR1‐activated CAFs could also induce SMAD2 phosphorylation in 19TT cells, whereas EVs from non‐activated CAFs did not have this effect (Figure 4c). To analyze the effect of TGF‐β‐EVs on TGF‐β signaling in real‐time, we utilized the (CAGA)_12_‐SMAD‐dependent EGFP transcriptional reporter assay, revealing that TGF‐β‐EVs triggered an early response in SMAD transcriptional response (Figure 4d). Whereas the effect of TGF‐β‐EVs on the (CAGA)_12_‐EGFP reporter remained relatively stable after 24 h, TGF‐β ligand stimulation continuously induced the (CAGA)_12_‐EGFP reporter over 48 h (Figure 4d). In contrast, TSG6‐depleted EVs failed to induce the (CAGA)_12_‐EGFP reporter, and TGF‐β‐EVs with either THBS1 or TGFBI depletion promoted the reporter activity only minimally (Figure 4e). When SMAD3 was depleted to abrogate TGF‐β/SMAD signaling in CAFs, the CAF activation effect of TGF‐β‐EVs was blocked (Figure 4f; Figure S6c, Supporting Information), indicating that the CAF activation effect of TGF‐β‐EVs is mainly mediated by TGF‐β/SMAD signaling. In addition, EVs derived from 19TT SMAD3‐depleted cells failed to activate TGF‐β signaling compared with TGF‐β‐EVs from 19TT vector‐transduced control cells (Figure 4g), indicating that SMAD3 in donor 19TT cells is required for the production of functional EVs.

As we observed that con‐EVs and TGF‐β‐EVs derived from CAFs did not affect the proliferation or migration of MDA‐MB‐231 cells, we investigated whether con‐EVs and TGF‐β‐EVs from CAFs affect TGF‐β signaling in MDA‐MB‐231 cells. Consistent with the lack of function of CAF‐derived EVs on cancer cells, the CAF‐derived EVs could not induce the TGF‐β signaling in MDA‐MB‐231 cells (Figure S6d, Supporting Information).

### 2.5 TSG6 and THBS1 are Associated with the Surface of CAF‐Derived EVs and Essential for EV‐Mediated TGF‐β Signaling

To investigate the potential cellular origins of TSG6 and THBS1 in EVs, these proteins were fluorescently labeled in cells alongside the tetraspanin CD63. The THBS1 was co‐localized with CD63 in the perinuclear region in non‐treated cells, while THBS1 was dispersed and highly co‐localized with the peripheral multivesicular bodies (MVBs) after TGF‐β treatment (Figure S7a, Supporting Information). This suggests that THBS1 can be sorted into endosome‐derived EVs, also known as exosomes, through TGF‐β stimulation. In contrast, we observed no clear co‐localization between TSG6 and CD63 in both control and TGF‐β treatment conditions, implying the sorting of TSG6 into exosomes may not be the predominant pathway for TSG6‐positive EVs (Figure S7b, Supporting Information).

**Figure 5:**
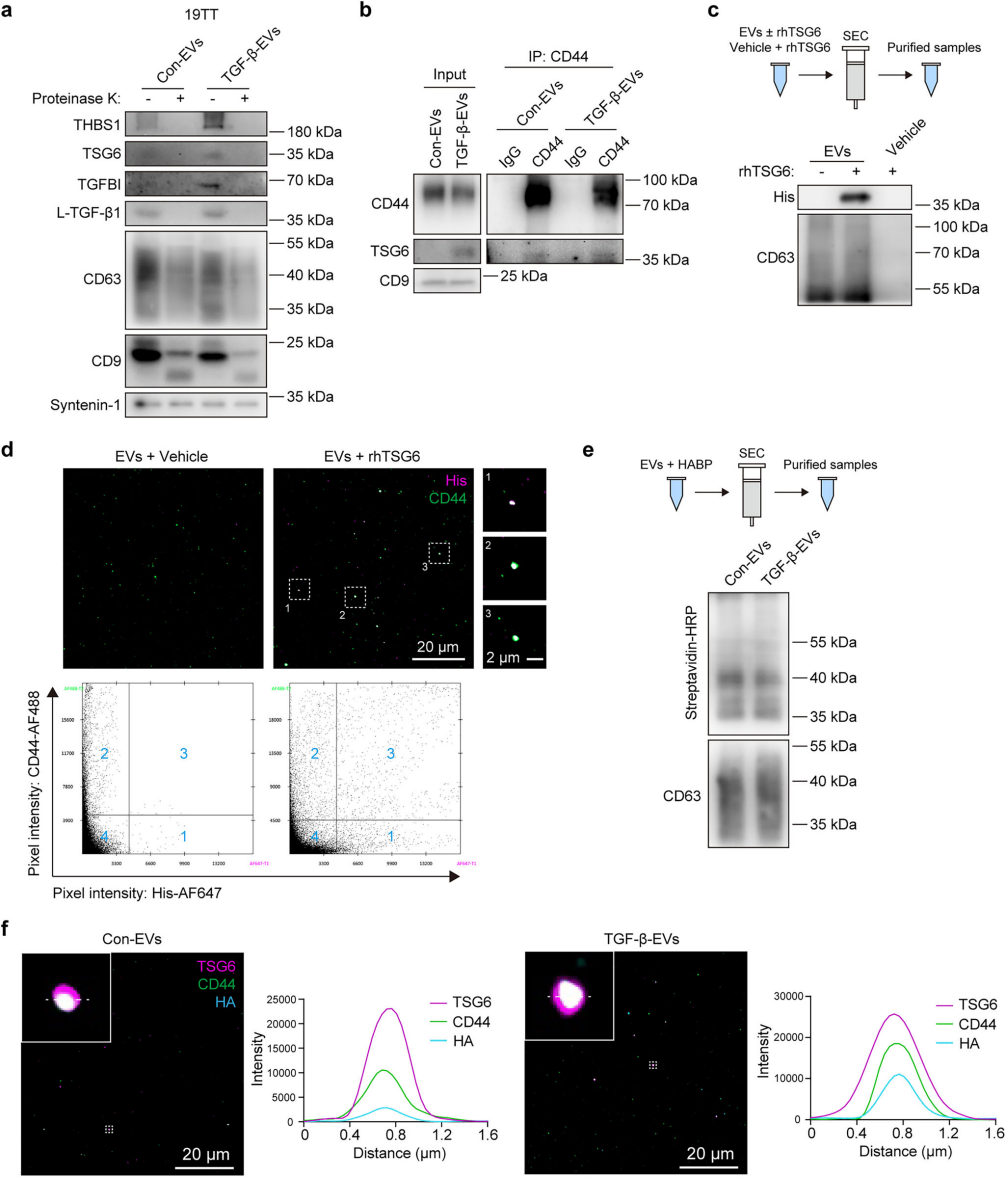
TSG6 and THBS1 are associated with the surface of CAF‐derived EVs. a) Western blot analysis of the indicated proteins from 19TT cell‐derived EVs with or without proteinase K treatment. Representative of *n* = 3 experiments. b) Co‐immunoprecipitation assay demonstrating the interaction between TSG6 and CD44 in 19TT cell‐derived EVs. IgG2b, negative control. Representative of *n* = 3 experiments. c) Western blot analysis of the interaction of recombinant human TSG6 (rhTSG6) with 19TT cell‐derived EVs. The top panel shows a schematic of the incubation of rhTSG6 with EVs, followed by EV purification. Representative of *n* = 3 experiments. d) Super‐resolution microscopy (SRM) imaging of His‐rhTSG6 and CD44 on EV surface. The scale bars represent 20 µm, and 2 µm in the magnified region. Representative double positive EVs from rhTSG6‐coated EVs are shown in the right panel. The bottom panel shows the co‐localization analysis. The area 1 represents the His‐rhTSG6‐AF647 single positive pixels. The area 2 represents the CD44‐AF488 single positive pixels. The area 3 represents the double positive pixels. The area 4 represents the background. The same threshold is applied to both conditions. Representative of *n* = 3 experiments. e) Western blot analysis of 19TT cell‐derived EVs for levels of hyaluronan (HA) using biotinylated hyaluronan bind protein (HABP). The top panel shows a schematic of the incubation of HABP with EVs, followed by EV purification. Representative of *n* = 3 experiments. f) SRM imaging of endogenous TSG6, CD44, and HA on con‐EV and TGF‐β‐EV surface. The scale bar represents 20 µm. Magnified regions represent triple positive EVs. Co‐localization analyses of magnified triple positive EVs are shown. Representative of *n* = 3 experiments.

TSG6, THBS1, and TGFBI are secreted proteins with high abundance in ECM,^[^ ^31^, ^32^, ^33^ ^]^ and their expression can be induced by TGF‐β (Figure 3g). It has been reported that TSG6 interacts with CD44,^[^ ^34^ ^]^ THBS1 interacts with CD47 and multiple β1 integrins,^[^ ^32^, ^35^ ^]^ and TGFBI also interacts with multiple integrins.^[^ ^36^ ^]^ CD44, CD47, and several integrins were identified among EV proteins by MS. Of note, TSG6 can also interact with THBS1.^[^ ^37^ ^]^ Therefore, they are likely to interact with the EV surface in the extracellular space, and from a large interactome on EV surface. To determine whether TSG6, THBS1, and TGFBI are present on the EV surface from 19TT cells, we performed surface labeling of these proteins, with CD63 labeling as a positive control, and used super‐resolution microscopy (SRM; 120 nm lateral resolution) to detect the fluorescence signal. The fluorescence signal of TSG6, THBS1, TGFBI, and CD63 was detected in both con‐EVs and TGF‐β‐EVs (Figure S8a, Supporting Information). The same labeling procedure was applied to phosphate‐buffered saline (PBS) as a negative control, and no fluorescence signal was detected, indicating there was no fluorochrome‐conjugated antibodies contamination in the labeled EVs after EV purification (Figure S8b, Supporting Information). Furthermore, 1% NP‐40 detergent was used to lyse the labeled EVs to exclude the possibility of the existence of antibody aggregates.^[^ ^38^ ^]^ Very little to no signal was detected in the EV samples treated with 1% NP‐40, indicating that the detected signals were indeed from the EVs (Figure S8b, Supporting Information). Next, we used proteinase K to cleave the surface proteins on EVs and then labeled the EVs with antibodies and MemGlow, a fluorogenic membrane probe. Most of the EVs detected by SRM were MemGlow‐positive, while very few were positive for TSG6, THBS1, and TGFBI (Figure S9a, Supporting Information), indicating that TSG6, THBS1, and TGFBI were cleaved by proteinase K on the EV surface. Almost no signal was detected in PBS negative control and 1% NP‐40‐treated EVs (Figure S9b, Supporting Information). Furthermore, we detected the protein levels of TSG6, THBS1, and TGFBI from total EVs lysates, with or without proteinase K pre‐treatment. Most of the TSG6, THBS1, and TGFBI were removed by proteinase K (**Figure** **5**a), suggesting that most of the TSG6, THBS1, and TGFBI were presented on the EV surface. The tetraspanins CD63 and CD9 were partially cleaved by proteinase K. In contrast, intravesicular protein Syntenin‐1 was almost unaffected^[^ ^39^ ^]^ (Figure 5a).

It is known that TSG6 interacts with CD44 and forms complexes with the polymer hyaluronan (HA) and CD44 on the cell surface.^[^ ^34^ ^]^ TSG6 also mediates the cross‐linking of HA and forms oligomer structures with multiple TSG6 proteins.^[^ ^40^ ^]^ We validated the interaction between TSG6 and CD44 in 19TT CAF cells using Co‐IP assay (Figure S9c, Supporting Information). Next, we analyzed CD44 expression in EVs and confirmed its interaction with TSG6 in EVs (Figure 5b). Furthermore, we incubated EVs with recombinant human TSG6 (rhTSG6) with a C‐terminal 10‐His tag. After SEC purification, we observed strong His signal, indicated that rhTSG6 can bind to EVs (Figure 5c). SRM was performed to detect the signal of CD44 and rhTSG6 on rhTSG6‐coated EVs. We found that the rhTSG6 was highly co‐localized with CD44^+^ EVs (Figure 5d). The rhTSG6 single positive EVs were also detected (Figure 5d), indicating that TSG6 may also bind to other receptors or that there may be partial nonspecific binding between TSG6 and EVs.

To examine whether HA was present on the EV surface, as HA can bind to TSG6 and CD44,^[^ ^34^ ^]^ we labeled EVs with HA‐binding protein (HABP). The presence of HA was detected in both con‐EVs and TGF‐β‐EVs, as indicated by the HABP signal from EV lysates (Figure 5e). To investigate whether HA co‐localized on EV surface with TSG6 and CD44, we labeled the HA, TSG6, and CD44 on EV surface. The TSG6^+^ CD44^+^ HA^+^ EVs were observed, suggesting that TSG6 and HA can form complexes with CD44 on the EV surface (Figure 5f). Next, we treated EVs with hyaluronidase to remove HA from their surface and incubated EVs with rhTSG6. The binding of rhTSG6 to hyaluronidase‐pretreated EVs was reduced compared to non‐treated EVs, suggesting that TSG6 can bind to HA on the EV surface (Figure S9d, Supporting Information).

As a high level of TSG6 was detected on the surface of EVs, we examined whether the soluble TSG6 has a similar effect as EV surface‐associated TSG6 by treating cells with different concentrations of soluble rhTSG6. The rhTSG6 treatment did not induce either TGF‐β signaling or CAF activation (Figure S10a,b, Supporting Information), whereas rhTSG6 potentiated the ability of TGF‐β‐EVs to activating TGF‐β signaling (Figure S10c, Supporting Information), suggesting that the EV surface is a crucial platform for TSG6 having its function on TGF‐β signaling. Next, we treated cells with rhTSG6‐coated EVs, but these EVs did not show a difference in function compared to con‐EVs in terms of inducing TGF‐β signaling or CAF activation (Figure S10d,e, Supporting Information). A similar effect was observed on rhTHBS1‐coated EVs (Figure S10f,g, Supporting Information). However, when we coated EVs with both rhTSG6 and rhTHBS1, these EVs partially mimicked the effect of TGF‐β‐EVs of inducing TGF‐β signaling pathway (Figure S10h, Supporting Information). In addition, we validated the interaction between TSG6 and THBS1 using recombinant proteins (Figure S10i, Supporting Information), suggesting that TSG6 and THBS1 may stabilize each other on the EV surface. These results indicate that both surface‐associated TSG6 and THBS1 play a pivotal role for EV function.

### 2.6 TGF‐β‐EVs Potentiate TGF‐β Signaling in a Contact‐Dependent Manner by Inducing Receptor Clustering

Since EV uptake facilitates the delivery of diverse cargo and influences cellular biological response, we examined EV internalization in CAF cells using PKH67‐labeled CAF EVs. To generate a single‐cell suspension and remove potential cell surface‐associated EVs,^[^ ^41^ ^]^ cells were trypsinized prior to analysis by flow cytometry. EV internalization in 19TT CAFs was minimal within 2 and 4 h, particularly in comparison to the higher EV uptake observed in MDA‐MB‐231 cells (Figure S11a, Supporting Information). Poor internalization of CAF‐derived EVs was also observed in CD8^+^ T cells (Figure S11b, Supporting Information). However, fluorescence microscopy imaging of CD8^+^ T cells incubated with PKH67‐labeled EVs showed that these EVs were associated with the cell membrane of T cells (Figure S11c, Supporting Information). A PBS procedural control with only PKH67 dye showed a virtual absence of PKH67 dye aggregates after SEC. 3D reconstruction imaging of CD8⁺ T cells incubated with PKH67‐labeled EVs further confirmed the association of EVs with the cell surface (Video S1, Supporting Information).

**Figure 6:**
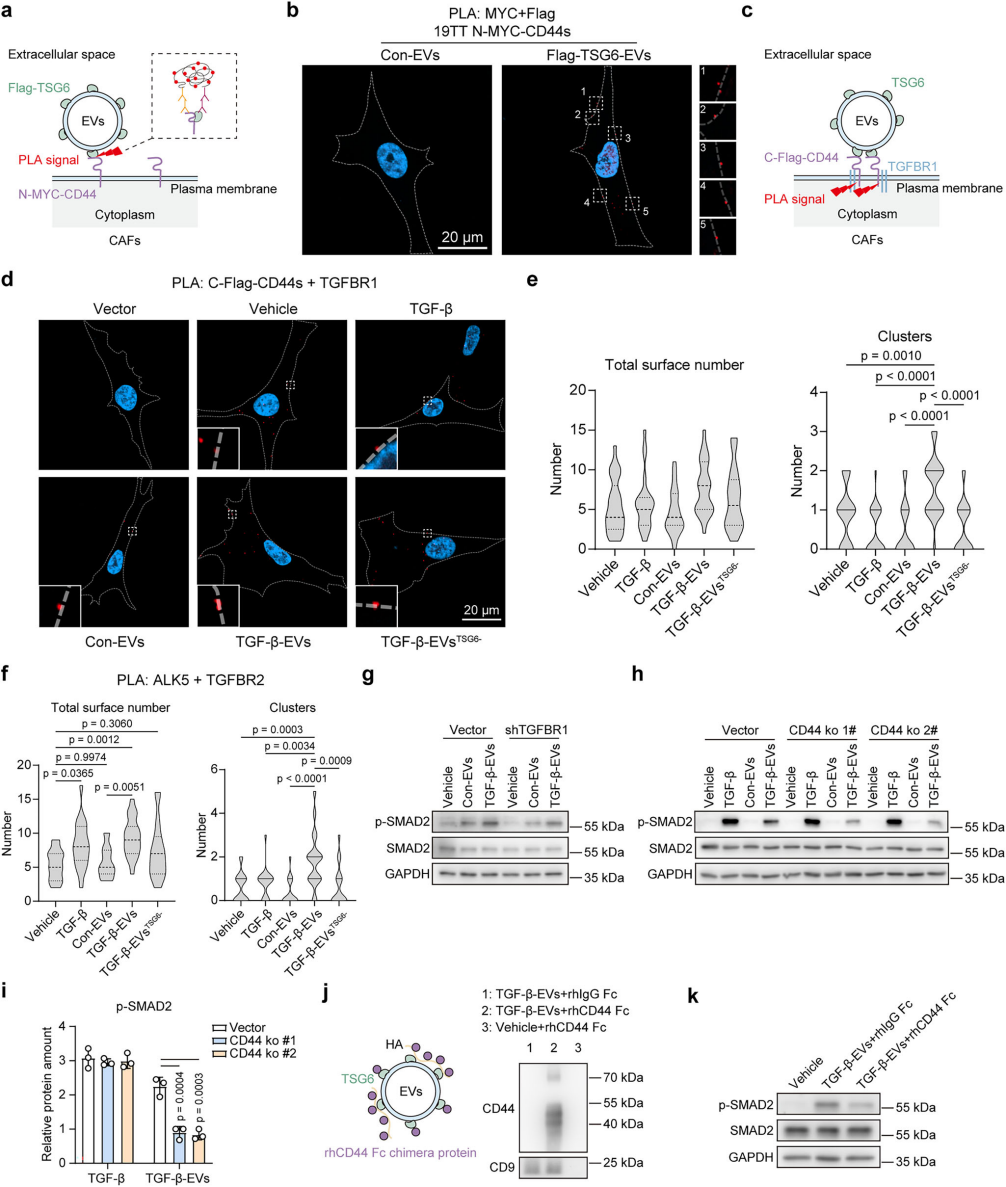
TGF‐β‐EVs potentiate TGF‐β signaling in a contact‐dependent manner. a) Schematic illustrating the detection of TSG6‐CD44s‐mediated EV docking on target cells using proximity ligation assay (PLA). PLA allows for detection of protein–protein interaction with single molecule resolution; schematic diagram is indicated on the right. b) Super‐resolution microscopy (SRM) imaging of PLA targeting MYC and Flag tags in N‐MYC‐CD44s overexpressing 19TT cells. Cells were treated with control EVs or EVs carrying Flag‐TSG6 for 2 h. The representative PLA signals on the cell surface are shown in the right panel. The scale bar represents 20 µm. Representative of *n* = 3 experiments. c) Schematic of PLA detecting the interactions between C‐Flag‐CD44s and TGFBR1 on the cell surface and the clusters of PLA signals (≥2 PLA signals within 1 µm) induced by EVs. d) SRM imaging of PLA targeting C‐Flag‐CD44s and TGFBR1 in 19TT cells. Cells were incubated with indicated treatments for 2 h. Magnified representative PLA signals on the cell surface are shown. The scale bar represents 20 µm. Representative of *n* = 3 experiments. e) Quantification of total PLA signals per cell and clustering events on the cell surface per cell from (d). More than 20 cells per condition were used for quantification. Representative of *n* = 3 experiments. f) Quantification of total PLA signals targeting TGFBR1 and TGFBR2 on the cell surface per cell and clustering events on the cell surface per cell. 19TT cells were incubated with indicated treatments for 2 h. More than 20 cells per condition were used for quantification. Representative of *n* = 3 experiments. g) Western blot analysis showing the effect of shRNA‐mediated TGFBR1 depletion on EV‐mediated TGF‐β signaling in 19TT cells. GAPDH, loading control. Representative of *n* = 3 experiments. h,i) Western blot analysis of the effect of TGF‐β and 19TT cell‐derived EVs on TGF‐β signaling in CRISPR‐Cas9‐mediated CD44 knockout 19TT cells. GAPDH, loading control. Representative of *n* = 3 experiments. Quantification of the relative protein level of p‐SMAD2 is shown. Means ± SD, *n* = 3 independent experiments, one‐way ANOVA with Dunnett's test. j) Left: Schematic of coating TGF‐β‐EVs with rhCD44‐Fc chimera protein or rhIgG‐Fc chimera protein control. The TGF‐β‐EVs were purified using SEC after incubation. Right: The binding of rhCD44‐Fc with TGF‐β‐EVs was validated using western blot. Representative of *n* = 2 experiments. k) Western blot analysis showing the effect of TGF‐β‐EVs incubated with rhCD44‐Fc or isotype control rhIgG‐Fc on TGF‐β signaling in 19TT cells. GAPDH, loading control. Representative of *n* = 3 experiments.

To investigate whether EV surface‐associated proteins can mediate the binding of EVs to cell receptors, we performed proximity ligation assay (PLA), which enables the highly specific detection of protein interactions within a distance of 40 nm.^[^ ^42^ ^]^ We constructed N‐terminal MYC‐tagged CD44‐overexpressing CAF cells as EV‐target cells, and isolated con‐EVs and Flag‐TSG6 EVs from empty vector‐transduced CAFs and Flag‐TSG6‐overexpressing CAFs, respectively (Figure S11d–f, Supporting Information). A PLA assay was performed on MYC‐CD44 and Flag‐TSG6 (**Figure** **6**a), which aimed to discriminate the detection of the interaction between endogenous TSG6 and CD44 on the EV surface and the potential interaction between endogenous soluble TSG6 and CD44 on the cell surface. Detectable PLA signals on the cell membrane of Flag‐TSG6 EV‐treated cells suggests that EVs can dock to the cell surface through protein–protein interactions (Figure 6b).

Given the limited CAF‐derived EV uptake in CAFs and CD8^+^ T cells, and the induction of TGF‐β signaling within 2 h, we hypothesized that CAF‐derived EVs primarily mediate TGF‐β signaling at the cell membrane through interactions with cell surface receptors. As such, this contact‐dependent mechanism could be central to the function of TGF‐β‐EVs. Furthermore, as TSG6 on the EV surface can interact with CD44 on the target cell surface (Figure 6b), and CD44 is known to interact with TGFBR1 in their cytoplasmic domains,^[^ ^43^ ^]^ we speculated that the upregulated TSG6 on TGF‐β‐EVs may recruit multiple CD44 receptors and induce the clustering of TGF‐β receptors with CD44 at the EV‐cell contact zone, forming signalosome structures, which can promote downstream signaling.^[^ ^20^ ^]^ To investigate our hypothesis, we constructed C‐terminal Flag‐tagged CD44‐overexpressing 19TT cells (Figure S11g, Supporting Information), and performed the PLA assay targeting TGFBR1 (not present in EV protein) and Flag‐CD44. The *z*‐position was selected based on the largest cross‐sectional area of the cell nucleus, which served as a consistent and representative focal plane. As the CAFs are morphologically flat, minimal variation in PLA signals was observed across different *z*‐stacks (Videos S2 and S3, Supporting Information). Positive PLA signals were detected in close proximity to the cell membrane and in the cytoplasm using SRM, while no signal was detected in 19TT CAFs transduced with the empty vector, indicating TGFBR1 can indeed interact with CD44 in 19TT CAFs (Figure 6d). To avoid selection bias, imaging fields were randomly chosen across different regions of each glass slide. The total surface PLA signals and the number of clusters on cell membrane were counted. A cluster was defined as ≥2 PLA signals within 1 µm. We found that TGF‐β and EV treatments did not affect the number of total surface signals. However, TGF‐β‐EV treatment increased the number of clusters on the cell surface compared to other conditions (Figure 6e), indicating that TGF‐β‐EV can promote the clustering of CD44 and TGFBR1 complexes. The effect of TGF‐β‐EVs was abolished when TSG6 was depleted, suggesting an essential role of TSG6 in this EV‐mediated receptor clustering mechanism. Similar PLA assays targeting heterodimeric complexes between TGFBR1 and TGFBR2 were conducted in CAFs under the previous treatment conditions (Figure S12a, Supporting Information). The number of PLA signals in close proximity to the cell membrane was increased in TGF‐β and TGF‐β‐EV treatment groups (Figure 6f). TGF‐β‐EVs enhanced the clustering of PLA signals targeting TGFBR1 and TGFBR2 in close proximity to the cell membrane, whereas TGF‐β did not have a significant effect on the surface distribution of PLA signals (Figure 6f). Furthermore, TGF‐β‐EVs increased the number of surface PLA signals targeting TGFBR2 and CD44 compared to vehicle‐treated cells, and also enhanced the clustering of TGFBR2 and CD44 complexes (Figure S12b,c, Supporting Information). This suggests that TGF‐β‐EVs promote the formation of CD44 and TGF‐β receptor complexes, as a direct interaction between TGFBR2 and CD44 is unlikely,^[^ ^43^ ^]^ and TGF‐β‐EVs facilitate the clustering of receptor complexes.

### 2.7 TGFBR1 and CD44 on the Cell Surface are Essential for TGF‐β‐EV‐Mediated TGF‐β Signaling

To further examine the role of TGF‐β receptors and CD44 in TGF‐β‐EV‐mediated TGF‐β signaling, we tested EVs on 19TT cells with shRNA‐mediated TGFBR1 knockdown (Figure S11h, Supporting Information). The ability of TGF‐β‐EVs to induce TGF‐β signaling and promote CAF activation was inhibited (Figure 6g; Figure S11i, Supporting Information), indicating that TGFBR1 is required for the effect of TGF‐β‐EVs on CAFs. Moreover, we tested EVs on CRISPR‐Cas9‐mediated CD44 knockout 19TT cells (Figure S11j, Supporting Information), where CD44 depletion did not affect SMAD2 phosphorylation in response to TGF‐β ligand stimulation. However, it significantly reduced SMAD2 phosphorylation induced by TGF‐β‐EVs (Figure 6h,i). This suggests that the effect of TGF‐β‐EVs on TGFBR signaling is dependent on CD44 and that TGF‐β‐EVs mediate signaling through a mechanism distinct from that of soluble TGF‐β ligands. To further investigate the role of EV‐CD44 interactions at the cell surface in EV‐mediated TGF‐β signaling, we coated TGF‐β‐EVs with an excess of recombinant human CD44‐Fc chimera protein (rhCD44‐Fc) to block CD44 binding sites on their surface. Recombinant human IgG1 Fc protein (rhIgG‐Fc) coating was used as a control. We then validated the binding of rhCD44‐Fc with TGF‐β‐EVs (Figure 6j), and treated 19TT cells with rhIgG‐Fc‐coated TGF‐β‐EVs and rhCD44‐Fc‐coated TGF‐β‐EVs. We found that rhIgG‐Fc‐coated TGF‐β‐EVs still upregulated the expression of p‐SMAD2. In contrast, rhCD44‐Fc‐coated TGF‐β‐EVs decreased the level of p‐SMAD2 compared to rhIgG‐Fc‐coated TGF‐β‐EVs (Figure 6k). The rhCD44‐Fc coating on TGF‐β‐EVs also inhibited their ability to induce the formation of TGFBR1 and TGFBR2 receptor clusters (Figure S13a,b, Supporting Information). These results indicate that CD44 on the cell surface plays a critical role in EV‐mediated TGF‐β/SMAD signaling.

To explore whether TGF‐β‐EVs induced TGF‐β/SMAD signaling by clustering TGF‐β receptors is independent of TGF‐β ligands or not, we treated the cells with TGF‐β neutralizing antibody‐1D11. We found that the TGF‐β/SMAD signaling inducing effect by TGF‐β‐EVs was blocked by 1D11 (Figure S11k, Supporting Information), indicating the effect of TGF‐β‐EVs on TGF‐β signaling requires the presence of active TGF‐β. When the kinase activity of TGFBR1 was inhibited by SB505124 treatment, the stimulatory effect of TGF‐β‐EVs was also blocked (Figure S11k, Supporting Information), consistent with the previous result that TGFBR1 depletion inhibited TGF‐β‐EV‐mediated TGF‐β signaling. Next, we examined the effect of EVs on TGF‐β signaling in a serum‐free medium to determine whether the low levels of TGF‐β ligands in the serum are required for EV‐mediated TGF‐β signaling. Notably, TGF‐β‐EVs still activated TGF‐β signaling in CAFs (Figure S11l, Supporting Information), indicating that this mechanism does not rely on the TGF‐β ligands from serum. The proteomics result showed that L‐TGF‐β1 was present in the EV protein. By treating EVs with proteinase K, we found that the majority of L‐TGF‐β1 was located on the surface of EVs and exhibited similar expression levels in con‐EVs and TGF‐β‐EVs (Figure 5a). When TGF‐β‐EVs were pre‐incubated with 1D11, followed by purification using SEC. The ability of these 1D11 antibody‐coated EVs to induce TGF‐β signaling was markedly reduced (Figure S11m, Supporting Information), demonstrating that latent TGF‐β1 on the EV surface is important for TGF‐β‐EV function. Furthermore, we treated cells with various low concentrations of TGF‐β and found that TGF‐β‐EVs can indeed enhance the potentiating effect of the low TGF‐β concentrations on SMAD signaling (Figure S11n, Supporting Information).

**Figure 7:**
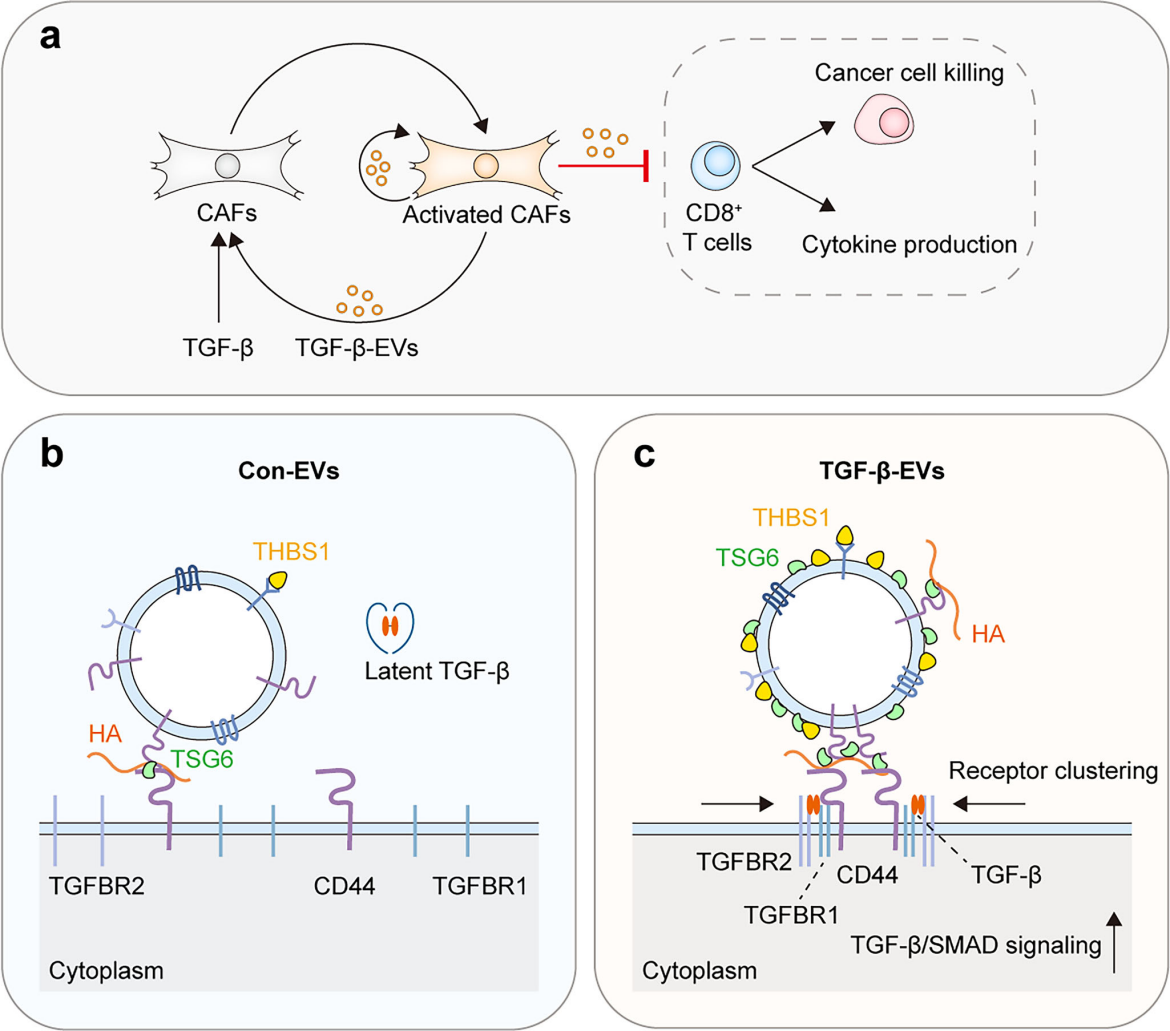
Schematic of a working model for TGF‐β‐activated CAF‐derived EVs in the tumor microenvironment. a) EVs derived from TGF‐β‐activated CAFs (TGF‐β‐EVs) promote and sustain CAF activation and impair the cancer cell‐killing ability and cytokine production of CD8^+^ T cells. b,c) Proposed mechanism of a contact dependent process in which TGF‐β‐EVs regulate cell signaling through increased expression of EV surface‐associated (coronal) proteins, i.e., tumor necrosis factor‐inducible gene 6 protein (TSG6), which is a ligand for co‐receptor CD44 and interacts with hyaluronan (HA), and thrombospondin‐1 (THBS1), which is an activator of latent TGF‐β. TGF‐β‐EV‐mediated CD44/TGFBR clustering on target cells enhances TGF‐β/SMAD signaling compared with con‐EVs.

Taken together, TGF‐β‐EVs and their surface‐associated proteins can potentiate TGF‐β signaling in a contact‐dependent manner by promoting the clustering of CD44 and TGF‐β receptors on the 19TT cell membrane (**Figure** **7**).

## 3 Discussion

In this study, we revealed a critical function by which EVs derived from TGF‐β‐activated CAFs modulate the TME. Compared with normal CAF‐derived EVs, TGF‐β‐EVs can promote CAF activation, and induce immunosuppression of CD8^+^ T cells by potentiating TGF‐β signaling in target cells via EV surface‐associated proteins. Furthermore, we discovered an underlying mechanism of EV‐mediated cell signaling using CAF‐derived EVs as a model; EV surface‐associated proteins facilitate the docking of EVs to the cell membrane via cell receptors and induce receptor clustering, thereby increasing the sensitivity of cells to TGF‐β ligands and promoting TGF‐β/SMAD signaling.

EV surface‐associated proteins and other components provide a highly interactive and dynamic surface area on EVs, facilitating EV interactions with the extracellular environment and cells. We showed that TGF‐β significantly upregulated the total amount of protein uploaded with EVs. By performing quantitative proteomics with an equal number of EVs measured by MRPS, we revealed that most differentially expressed proteins were upregulated in TGF‐β‐EVs, with a high proportion consisting of secreted proteins. Several of the top upregulated proteins (with high expression in TGF‐β‐EVs) were selected for further analysis, including TSG6, THBS1, and TGFBI. These proteins, highly abundant in the extracellular space, can interact with ECM components.^[^ ^32^, ^34^, ^36^, ^44^ ^]^ To assess whether they are internal or present on the EV surface, we treated EVs with proteinase K to cleave surface proteins and detected their surface expression and total amount using SRM and western blot analysis, respectively. Most of the EV surface protein expression and total protein level was removed by proteinase K, indicating that these proteins are predominantly localized on the EV surface. Since TSG6 can interact with CD44,^[^ ^34^ ^]^ we labeled the EV surface with TSG6, CD44, and their interacting ECM component HA, and we demonstrated that these three components could be present on the same EV. This suggests that these EV surface‐associated proteins can form complexes through interactions with their receptors and ECM components.

EV surface‐associated proteins may originate from their parental cell, be acquired from the extracellular space, or both. Staining CAFs for TSG6 revealed no clear co‐localization with MVBs, even after TGF‐β treatment, suggesting that exosome packaging is not the primary source of TSG6‐positive EVs. In contrast, THBS1 was sorted into MVBs following TGF‐β treatment and subsequently transported to the cell periphery. However, whether THBS1 is incorporated into intraluminal vesicles (ILVs), attached on the surface of ILVs, or both, remains to be explored. Given that the EV surface is in direct contact with the extracellular space, EVs can function as molecular sponges, adsorbing proteins and matrix components. By incubating EVs with recombinant TSG6 and THBS1, we showed that these recombinant proteins can bind to EVs, suggesting the extracellular space as a critical source of surface‐associated EV cargo and a key contributor to EV functional properties.

We further revealed the mechanism by which CAF‐derived EV surface‐associated proteins can regulate CAF and T cell behavior in a contact‐dependent manner. Our finding contrasts with a previous study that showed CAF‐derived EVs induce TGF‐β signaling in target cancer cells dependent on EV uptake.^[^ ^45^ ^]^ The TSG6 on the surface of TGF‐β‐EVs can interact with CD44 and HA, and mediate the docking of EVs on the cell surface. The upregulated TSG6 on TGF‐β‐EVs can bind multiple CD44 receptors that recruit TGF‐β receptors, forming signalosomes that promote TGF‐β signaling. CD44 and TGF‐β receptors on the cell surface are essential for TGF‐β‐EV‐mediated TGF‐β signaling, whereas TGF‐β signaling induced by soluble TGF‐β ligands does not depend on CD44. Of note, TSG6 is only present in CAF‐derived EVs, but not in MDA‐MB‐231 cancer cells. Therefore, the TSG6/CD44‐mediated mechanism is likely unique to CAF‐derived EVs compared with those from MDA‐MB‐231 cells.

Although the upregulated EV surface‐associated TSG6 is essential for this process, other upregulated proteins on the EV surface also contribute significantly to this process. For example, when we depleted THBS1 on EVs, the effect of TGF‐β‐EVs on inducing TGF‐β signaling was nearly abolished, and the subsequent CAF activation effect and CD8^+^ T cell suppressive effect by TGF‐β were inhibited. In addition, the rhTSG6‐coated EVs cannot mimic the effect of TGF‐β‐EVs on activating TGF‐β signaling. When the EVs were coated with both rhTSG6 and rhTHBS1, the effect of TGF‐β‐EVs on activating TGF‐β signaling could be partially rescued. The interaction between TSG6 and THBS1 may play an important role in their mutual stabilization on the EV surface. As THBS1 can bind with multiple receptors including CD47 and integrins,^[^ ^35^ ^]^ we speculate that THBS1 has a synergistic effect on the docking of EVs to the cell membrane via its receptors and stabilizes EVs on the cell surface. When the EVs dock with cells, the EV membrane enables lateral diffusion and facilitates the sorting of multiple EV surface‐associated proteins to the EV‐cell contact zone, where they interact with their receptors.^[^ ^20^ ^]^

EV‐mediated TGF‐β signaling depends on the presence of TGF‐β ligands, even at low levels. We found that the majority of L‐TGF‐β1 was on the EV surface, consistent with a previous study.^[^ ^46^ ^]^ We demonstrated that the EV‐mediated TGF‐β signaling did not rely on the TGF‐β ligands from serum and that the L‐TGF‐β1 on the EV surface was sufficient to promote EV‐induced TGF‐β signaling. THBS1 may play an important role in activating the latent TGF‐β ligands on EV surface.^[^ ^47^ ^]^ EV surface‐associated L‐TGF‐β1 may also bind to integrin αvβ8 on target cells, where αvβ8 redistributes the intrinsic flexibility of TGF‐β1 and exposes mature TGF‐β1 to TGFBR2 without release.^[^ ^48^ ^]^

The uptake of EVs is very limited in CAFs and CD8^+^ T cells, consistent with findings from a previous study.^[^ ^49^ ^]^ Therefore, the effect of TGF‐β‐EVs on CAFs and CD8^+^ T cells is primarily based on a contact‐dependent manner. Following docking with the cell membrane, EVs can detach back into the extracellular space or undergo a “kiss‐and‐run” process.^[^ ^50^ ^]^ Although EV uptake in MDA‐MB‐231 and MCF‐7 cells was significant, the specific phenotype of these cancer cells was not altered by EVs. We speculate that the EVs on the surface of cancer cells may be transient and not remain long enough to induce receptor clustering before the rapid uptake. Moreover, the CAF‐derived EVs deliver various cargo to the cancer cells, leading to more complex mechanisms.

Multiple, interrelated factors shape the immunosuppressive landscape of the TME. Beyond CAF‐derived EVs, both tumor cells and CAFs secrete soluble mediators such as TGF‐β and release their own EV populations, which together influence immune and stromal cells to promote immunosuppression. In this study, we showed that TGF‐β activates CAFs and induces the release of EVs with immunosuppressive properties. However, the relative contribution of these TGF‐β‐activated CAF‐derived EVs compared with tumor cell‐derived EVs or soluble TGF‐β in vivo remains to be determined. We speculate that CAF‐derived EVs act as a downstream mediator that sustains or amplifies the immunosuppressive effects initially triggered by soluble TGF‐β. While soluble TGF‐β exerts transient and diffusible effects, EVs can deliver stable cargos such as proteins, RNAs, and lipids to specific immune targets, potentially resulting in more durable or localized immune modulation. Thus, CAF‐derived EVs may represent a mechanism through which TGF‐β signaling is stabilized and propagated within the TME. Future in vivo studies using systems to trace or inhibit EV secretion from CAFs selectively will be critical to clarify these interactions and define the specific contribution of CAF‐derived EVs to tumor‐associated immunosuppression.

CAFs are highly heterogenous in the TME, and distinct CAF subtypes may vary in their capacity to produce EVs and modulate immune responses. Identifying which CAF populations are primarily responsible for the release of immunosuppressive EVs will be crucial for developing selective therapeutic strategies. Such insights could enable the targeting of pro‐tumorigenic CAF subsets or their EV‐mediated signaling while preserving CAF populations with tumor‐restraining functions.

As EV surface‐associated proteins are derived from both the parental cell and the extracellular space, they can reflect the physiological and pathological states of diseases and hold significant promise for biomarker discovery and the development of robust, EV‐based noninvasive liquid biopsies. Moreover, our study expands the potential for biomedical applications and therapies based on EV surface proteins, through EV surface protein engineering or by loading functional proteins onto the EV surface for therapeutic purposes. These approaches could offer promising opportunities for targeted therapies, improving the delivery and modulation of therapeutic proteins.

## 4 Experimental Section

### Cell Culture and Reagents

HEK293T (CRL‐1573, RRID: CVCL_0063), MDA‐MB‐231 (CRM‐HTB‐26, RRID: CVCL_0062), MCF‐7 (HTB‐22, RRID: CVCL_0031), and BT‐474 (HTB‐20, RRID: CVCL_0179) cells were purchased from the American Type Culture Collection (ATCC). The human telomerase reverse transcriptase (hTERT)‐immortalized human breast CAFs 19TT cells have been described^[^ ^51^ ^]^ and used in the previous study.^[^ ^52^ ^]^ HTERT‐immortalized human breast CAFs LACAF cells were obtained from Dr. Thomas A Hughes (University of Leeds, Leeds, UK) and have been previously described.^[^ ^53^ ^]^ HTERT‐immortalized human breast CAFs CAF2 cells were obtained from Dr. Akira Orimo (Juntendo University, Bunkyo, Japan) and Dr. Kristian Pietras (Lund University, Lund, Sweden) and have been previously described.^[^ ^54^, ^55^ ^]^ KPC3‐OVA cells were obtained from Dr. Ferry Ossendorp (Leiden University Medical Center, Leiden, Netherlands) and have been described in the previous study.^[^ ^26^ ^]^ BALB/c mouse primary mammary fibroblasts were purchased from CellBiologics (BALB‐5071), and cultured in a complete fibroblast medium (CellBiologics, M2267). BT‐474 cells were cultured in RPMI 1640 medium (Thermo Fisher Scientific, 21875034), and other cell lines and CAF cells were cultured in Dulbecco's modified Eagle medium (DMEM, Thermo Fisher Scientific, 41966029). The RPMI 1640 and DMEM medium were supplemented with 10% (v/v) fetal bovine serum (FBS, S1810, BioWest), 100 U mL^−1^ penicillin and 100 µg mL^−1^ streptomycin (Thermo Fisher Scientific, 15140163). All CAFs were routinely cultured in 0.1% gelatin‐coated (G1890, Sigma‐Aldrich) plates to avoid possible activation caused by physical rigidity.^[^ ^52^ ^]^ All cell lines were maintained in a 37 °C and 5% CO_2_ humidified incubator, tested routinely for mycoplasma contamination, and checked for authenticity by short tandem repeat profiling. Recombinant human TGF‐β3 (generous gift from Dr. A. Hinck, University of Pittsburgh, USA) was used at 2.5 ng mL^−1^ for cell treatment, unless otherwise specified. A selective small molecule TGFBR1 inhibitor SB505124 was used at a concentration of 1 µm. The information of other recombinant proteins is listed in Table S1 (Supporting Information).

### T Cell Isolation, Activation, and Culture

Peripheral blood of healthy donors was obtained via the Leiden University Medical Center healthy voluntary donor service (LuVDS). All healthy donors gave broad consent. The biomaterial and associated clinical data of all healthy donors collected in the LuVDS are released for research purposes only, after being approved by the internal review board. Human CD8^+^ T cells were isolated from the peripheral blood mononuclear cells (PBMCs) of healthy blood donors in accordance with the Declaration of Helsinki and the Dutch rules with respect to the use of human materials from volunteer donors. PBMCs were isolated by density gradient centrifugation with Histopaque‐1077 (Sigma, 10771), following the manufacturer's instructions. Briefly, fresh peripheral blood was collected in sodium heparin tubes and layered on top of the Histopaque‐1077 solution. After centrifugation at 400 *g* for 30 min at room temperature, the PBMCs in the opaque interface were transferred to a new tube and washed three times with phosphate‐buffered saline (PBS). CD8^+^ T cells were further purified by negative isolation using Dynabeads and an antibody mix targeting other cell types in PBMCs (Thermo Fisher Scientific, 11348D) according to the manufacturer's instructions. CD8^+^ T cells were cultured in Iscove's Modified Dulbecco's Medium (IMDM, Thermo Fisher Scientific, 21980032) supplemented with 8% (v/v) heat‐inactivated human serum (PAN‐Biotech, P40‐2701), 4 mm L‐Glutamine (Thermo Fisher Scientific, A2916801), 50 U mL^−1^ penicillin and 50 µg mL^−1^ streptomycin (Thermo Fisher Scientific, 15140163). CD8^+^ T cells were activated using Dynabeads Human T‐Activator CD3/CD28 beads (Thermo Fisher Scientific, 11131D). Activated CD8^+^ T cells were cultured with 5 ng mL^−1^ recombinant human IL‐2 (PeproTech, 200‐02). The isolation and activation of OT‐I CD8^+^ T cells have been described previously. The animal experiments were designed in accordance with the Code of Practice of the Dutch Animal Ethical Commission and were conducted in accordance with the regulations stated in project AVD11600202013796.^[^ ^26^ ^]^

### Plasmids

The cDNA fragments encoding full open reading frames of human TSG6 and CD44s were amplified by PCR from the cDNA of 19TT cells, and cloned into pLV‐cytomegalovirus (CMV)‐intra ribosomal entry site (IRES)‐neomycin (Neo) vectors. The short hairpin RNA (shRNA) constructs for the knockdown of *TSG6*, *THBS1*, *TGFBI*, *SMAD3*, and *TGFBR*1 were obtained from the Mission shRNA library of Sigma‐Aldrich. The puromycin resistance gene in the original constructs was replaced with the hygromycin resistance gene. Detailed information of shRNA constructs is provided in Table S2 (Supporting Information). Two knockout guides against human *CD44* were designed using the ChopChop webtool (https://chopchop.cbu.uib.no/) (Target sequences; 1: TCGCTACAGCATCTCTCGGACGG, 2: CTACAGCATCTCTCGGACGGAGG). The guides were cloned in the pLENTI‐CRISPR‐V2 plasmid. All DNA sequences of modified plasmids were verified by Sanger DNA sequencing.

### Lentiviral Packaging and Generation of Stable Cell Lines

A third‐generation lentiviral packaging system (pRSV‐Rev, pMDLg/pRRE and pCMV‐VSV‐G) was used for lentiviral production. Briefly, plasmids were transfected into HEK293T using polyethylenimine (PEI, Polysciences, 23966) for lentiviral production. Cell supernatants were collected at 48–72 h post‐transfection and filtered through 0.45 µm polyvinylidene fluoride (PVDF) membrane filters (Millipore, SLHVR33RB). To generate stable cell lines, cells were infected with lentiviral supernatants supplemented with an equal volume of fresh medium. After 24 h of infection, the cells were expanded and cultured in the corresponding antibiotics for selection.

### Preparation of Conditioned Medium and EV Isolation

EV‐depleted FBS was prepared using regular FBS filtered by 100 kDa Amicon ultra‐15 centrifugal filters (Millipore, UFC910024) at 3200 *g* in a swinging‐bucket rotor.^[^ ^56^ ^]^

Cells were seeded in 15 cm culture dishes with completed medium, and recombinant human TGF‐β3 (2.5 ng mL^−1^) or ligand buffer (vehicle control) was added to the cells for pre‐treatment the following day for 24 h. After pre‐treatment, the medium was discarded, and cells were washed with PBS. Medium supplemented with EV‐depleted FBS was added to the cells with treatments and collected after 48 h. At the time point of collection, the cells reached 95% confluency.

The conditioned medium was centrifuged at 300 × *g* for 5 min at 4 °C to remove cells. The supernatant was centrifuged at 2000 × *g* for 15 min, followed by 3200 × *g* for 15 min at 4 °C to remove cell debris. The supernatant was concentrated using 100 kDa Amicon ultra‐15 centrifugal filters at 3200 × *g* to a volume of 300 µL. The concentrated medium was transferred to 1.5 mL protein low‐binding tubes (Eppendorf, 0030108116). The filters were washed by adding an additional 200 µL PBS. The two volumes of medium were mixed to achieve a final volume of 500 µL for size exclusion chromatography (SEC). The EVs were further purified by SEC using 35 nm qEV columns (IZON, ICO‐35) and automatic fraction collector‐V2 (IZON, AFC‐V2) following the manufacturer's instructions. The buffer volume was set to 2.5 mL, and the purified collection volume was set to 2 mL in a single fraction on the AFC‐V2. EVs were separated from contaminating soluble proteins in this step. The EV samples were collected in protein low‐binding tubes, aliquoted, snap‐frozen, and stored at −80 °C.

### Nanoparticle Tracking Analysis (NTA)

The NTA was performed using a NanoSight NS300 equipped with a 488‐nm laser and NTA software v3.2. The samples were diluted in 0.22 µm filtered PBS to achieve concentrations that allowed the detection of 10 to 100 particles per frame during measurement. The temperature was set to 21 °C, and the camera level to 13, optimizing the visibility of small particles while minimizing background noise. The samples were loaded into a chamber using syringes and captured for 60 s for three repeats, and the images were captured at a rate of 25 frames s^−1^. The concentration and size distribution of EVs were analyzed with a detection threshold of 5.

### Microfluidic Resistive Pulse Sensing (MRPS)

The MRPS assay was performed using nCS1 (v0, Spectradyne) with C‐400 cartridges (specified size range: 65–400 nm). 1% (w/v) poloxamer‐188 (Alfa Aesar, J66087) in Dulbecco's Phosphate‐Buffered Saline (DPBS) (Corning, 21‐031‐CVR) was filtered through 0.05 µm Isopore filters (Whatman) before measurements. EV samples were diluted in DPBS with 0.05% (w/v) poloxamer‐188,^[^ ^57^ ^]^ and 6 µL of samples were loaded into cartridges for detection. Default settings were used for the data acquisition. The results were processed using nCS1 Viewer software (v2.5.0.297, Spectradyne). The false positive events were excluded by setting the peak filters as follows: Transit time < 60 µs and <100 µs based on Mold‐ID of the cartridge; Symmetry: 0.2–0.4; Signal to noise > 10; Diameter > 65 nm. A bin size of 10 nm was chosen to present the measured particle size distributions, and the reported concentrations included particles ranging from 70 to 400 nm.

### Immunoblotting (Western Blot)

For protein analysis of purified EV samples, the proteins were concentrated using trichloroacetic acid (TCA) precipitation as described.^[^ ^29^ ^]^ Sodium deoxycholate was added to the EV samples with a concentration of 2 mg mL^−1^. The ice‐cold 100% (w/v) TCA (Sigma‐Aldrich, 91230) was added to samples with a concentration of 20% and mixed immediately. The samples were incubated for 30 min at 4 °C, and centrifuged at 16 000 *g* for 10 min at 4 °C. The pellets were washed twice with 1 mL of pre‐cooled acetone and centrifuged as above. The protein pellets were air dried, resuspended in lysis buffer (10% [w/v] glycerol, 2% [w/v] SDS, and 60 mm Tris‐HCl [pH 6.8]) and heated at 95 °C for 5 min. For protein analysis of cell samples, the cells were washed with PBS and lysed with the lysis buffer described above, and the cell lysates were heated at 95 °C for 5 min. The protein concentrations were determined using the bicinchoninic acid (BCA) protein assay (Thermo Fisher Scientific, 23227), according to the manufacturer's instructions.

Proteins were separated by SDS‐polyacrylamide gel electrophoresis (PAGE) followed by transferring to 0.45 µm PVDF membranes (Millipore, IPVH00010). Membranes were blocked with 5% (w/v) non‐fat milk in TBST buffer (20 mm Tris‐HCl [pH 7.6], 150 mm NaCl, and 0.1% [v/v] Tween 20) for 1 h at room temperature. The membranes were incubated with primary antibodies diluted with 3% BSA in TBST buffer at 4 °C overnight and secondary antibodies diluted with 5% (w/v) non‐fat milk in TBST buffer at room temperature for 1 h. The membranes were washed four times with TBST buffer for 5 min after each incubation. The horseradish peroxidase (HRP) signals were detected with enhanced chemiluminescent (ECL) substrate (Bio‐Rad, 170‐5061) and ultra‐sensitive ECL substrate (Thermo Fisher Scientific, 34095) using a Bio‐Rad ChemiDoc imaging system. The results were further processed using Image Lab software. The antibodies used for western blot are listed in Table S3 (Supporting Information), except for anti‐phospho‐SMAD2.^[^ ^58^ ^]^

### RNA Extraction and Quantitative Real‐Time PCR

Total RNA was isolated from cells using NucleoSpin RNA kits (Macherey Nagel, 740955), according to the manufacturer's protocol. 1 µg RNA per sample was used for cDNA synthesis using RevertAid H Minus reverse transcription kit (Thermo Fisher Scientific, K1632). Quantitative PCR was performed using the GoTaq qPCR Master mix (Promega, A600X) on the CFX Connect Real‐Time PCR detection system (Bio‐Rad). 2^−ΔΔCt^ was calculated using the housekeeping gene glyceraldehyde 3‐phosphate dehydrogenase (GAPDH). The primer sequences used for RT‐qPCR are listed in Table S4 (Supporting Information).

### Collagen Gel Contraction Assay

The CAFs were mixed with collagen type I (Ibidi, 50204) and serum‐free medium to a concentration of 1 × 10^5^ cells mL^−1^ in 1.5 mg mL^−1^ collagen. A 0.5 mL aliquot of the mixture, along with treatments, was added to each well of 3% bovine serum albumin (BSA)‐precoated 24‐well plates. The plates were incubated at 37 °C for 30 min to solidify the gel. An additional 0.5 mL of DMEM medium containing treatments was then added to the gel. The plates were incubated at 37 °C for 3 days and imaged using the Bio‐Rad ChemiDoc imaging system. The surface area of the gels was quantified using ImageJ software.

### Cell Proliferation Assay

Cells were seeded into 96‐well plates, and treatments were added to the cells in the following days. The cells were kept culturing for 5 days. Each well was added with 20 µL of the CellTiter 96 AQueous One Solution reagent (Promega, G3581) in 100 µL culture medium, and the plates were incubated at 37 °C for 1 h. The measurements were performed every 24 h. The absorbance was read at 490 nm using a plate reader (PerkinElmer).

### IncuCyte Migration Assay

Cells were seeded in ImageLock plates (Essen BioScience, 4379). The following day, the medium was replaced with DMEM supplemented with 0.5% FBS for an additional 8 h of culture. The scratch wounds were generated with a WoundMaker tool (Essen BioScience), and the cells were washed with PBS. The cells were cultured in DMEM supplemented with 0.5% FBS and the migration was recorded in the Incucyte S3 (Sartorius) for 2–3 days.

### Killing Assay

BT‐474 mCherry cells were seeded in 96‐well plates with a density of 5000 cells per well. On the following day, the activated CD8^+^ T cells were seeded on the plates with an effector‐to‐target (E:T) ratio of 4:1, and the anti‐human epidermal growth factor receptor (HER)2 × anti‐CD3 epsilon (CD3E) bispecific antibody (100 ng mL^−1^, Absolute Antibody, bAb0183) and treatments were added with CD8^+^ T cells to the plates. The cells were cultured in the Incucyte S3 (Sartorius) and recorded for 2–3 days.

### Flow Cytometry

Treatments were added to T cells for 2 days. After 2 days, T cells were treated with a cell stimulation cocktail (Thermo Fisher Scientific, 00‐4970‐93) and a protein transport inhibitor cocktail (Thermo Fisher Scientific, 00‐4980‐03) for 6 h. T cells were washed with PBS and stained with Zombie Violet dye (BioLegend, 423113) in PBS at 4 °C for 30 min. For surface staining, cells were washed with FACS buffer (PBS supplemented with 0.1% [w/v] NaN3 and 5% [v/v] FBS) once, and then stained with fluorophore‐conjugated antibodies in FACS buffer at 4 °C for 30 min in dark. For intracellular staining, cells were washed after surface staining and fixed with 4% formaldehyde (Thermo Fisher Scientific, 20908) for 20 min at room temperature. After fixation, cells were washed once with PBS and resuspended with permeabilization buffer (PBS supplemented with 0.1% [w/v] saponin and 0.5% [w/v] BSA) for 10 min at room temperature. The fluorophore‐conjugated antibodies were added to the cells for intracellular staining at 4 °C for 30 min. The cells were washed three times with permeabilization buffer after staining. Samples were analyzed using Cytek Aurora flow cytometers, and the results were further processed using FlowJo v10.8. FlowClean was used to control the quality of results. The antibodies used for flow cytometry are listed in Table S5 (Supporting Information).

### Enzyme‐Linked Immunosorbent Assay (ELISA)

Treatments were added to T cells for 2 days. The supernatants from T cells were collected to detect IFN‐γ and TNF‐α. The Elisa experiments were performed using Human IFN‐γ (ALP) ELISA kits (MABTECH, 3420‐1A‐6) and Human TNF‐α (ALP) ELISA kits (MABTECH, 3512‐1A‐6) according to the manufacturer's instructions.

### T Cell Proliferation Assay

Cells were labelled with 1 µm CFSE dye (Thermo Fisher Scientific, C34554) in PBS for 8 min at room temperature in dark. Five times the staining volume of culture medium was added to the cells to remove free dye and incubated for 5 min. The cells were centrifugated and resuspended in fresh culture medium with indicated treatments. T cells were activated and cultured with the specified treatments. CFSE fluorescence from CD8⁺ T cells was measured after 2 days of coculture with BT‐474 cells.

### Co‐Immunoprecipitation (Co‐IP) Assay

Cells were harvested and washed with PBS. The cells were resuspended with RIPA buffer (50 mm Tris‐HCl [pH 7.4], 150 mm NaCl, 1% NP‐40, 0.5% sodium deoxycholate and 0.1% SDS) supplemented with protease inhibitor (Roche, 11836145001), and incubated on ice for 30 min. Cells were centrifuged at 14 000 *g* for 10 min at 4 °C. The supernatant was collected and incubated with CD44 primary antibody (Thermofisher, 14‐0441‐82) and IgG2b control (Thermofisher, 14‐4031‐85) overnight with gentle mixing. The following day, protein G agarose beads (Cytiva, 17‐0618‐02) were added to the antibody‐lysate mixture, and incubated at 4 °C for 2 h with gentle mixing. After incubation, beads were washed with wash buffer (0.1% NP‐40 in PBS) for 3 times. 1 × Loading buffer (2% SDS, 10% glycerol, 5% β‐mercaptoethanol, 0.01% bromphenol blue and 0.06 m Tris HCl [pH6.8]) was added to beads and heated at 95 °C for 5 min. For Co‐IP experiments involving EV samples, SEC‐purified EVs were incubated with the same volume of adjusted 2 × RIPA buffer (100 mm Tris‐HCl [pH 7.4], 150 mm NaCl, 2% NP‐40, 1% sodium deoxycholate, and 0.2% SDS) supplemented with protease inhibitor. The following procedures are identical to those used for Co‐IP experiments in cells.

### EV Surface Protein Shaving

SEC‐purified EVs were incubated with a final concentration of 25 µg mL^−1^ Proteinase K (Thermofisher, 25530031) for 1 h at 37 °C. A protease inhibitor was added to the solution after incubation. For western blot experiments, the EV protein was isolated using TCA protein precipitation as described above. For surface labeling of EVs, EVs were washed with 0.22 µm filtered PBS using EV spinners (Hansabiomed, HBM‐EVS100‐24), followed by antibody labeling.

### Immunofluorescence Assay and EV Labeling

Cells were seeded onto coverslips (1.5 H thickness, Roth, YX03.1) in 24‐well plates for immunofluorescent staining. After treatment, cells were fixed with 4% (v/v) formaldehyde (Thermo Fisher Scientific, 20908) for 15 min at room temperature. Cells were washed with PBS, permeabilized with 0.2% (v/v) Triton X‐100 for 10 min at room temperature, and then washed again with PBS. Cells were blocked with 3% (w/v) BSA in PBS for 30 min at room temperature, and incubated with primary antibodies overnight at 4 °C. After washing three times with PBS, cells were incubated with fluorophore‐conjugated secondary antibodies for 1 h at room temperature. All antibody dilutions were centrifuged at 16 000 *g* for 5 min at 4 °C to remove potential antibody aggregates. The slides were washed and mounted with antifade mounting medium with 4′,6‐diamidino‐2‐phenylindole (DAPI) (Vector Laboratories, H‐1500). Phalloidin conjugated with Alexa Fluor 488 (Thermo Fisher Scientific, A12379) was used for F‐actin labeling.

For EV antibody labeling, SEC‐purified EVs were incubated with primary antibodies for 2 h at room temperature. EVs were washed two times with EV spinners, and incubated with fluorophore‐conjugated secondary antibodies for 1 h at room temperature. After labeling, the EVs were washed two times and concentrated with EV spinners after labeling. For imaging, EV samples were diluted to avoid fluorescence signal overlap caused by EVs in close proximity. 3 µL per EV sample was used for imaging. Labeled EVs were incubated with 1% NP‐40 detergent and imaged within 3 min as a negative control.

Fields were randomly chosen across different regions of each slide. Samples were imaged using Zeiss LSM 900 with objective Plan‐Apochromat 63x/1.4 Oil DIC M27, and the Airyscan detector was used for super‐resolution imaging which achieves a lateral resolution of up to 120 nm. Images were processed using ZEN v3.7.

### EV Labeling and Uptake Assay

EVs were labeled with PKH67 dye (Sigma‐Aldrich, MINI67) according to the manufacturer's instructions with slight modifications. The purified EV samples after SEC were spun down using ultracentrifugation at 120 000 *g* for 90 min at 4 °C, and the EVs were resuspended in Diluent C. The dye solution was prepared by diluting the PKH67 dye with an equal volume of Diluent C to 4 µm. The EV suspension was added to the dye solution, mixed by gentle pipetting, and incubated at room temperature for 5 min. The dye was quenched with 1% BSA in 0.22 µm filtered PBS and incubated for 1 min. The labeled EVs were purified with SEC and concentrated with EV‐spinners. The labeled EVs were incubated with target cells at the indicated time points.

### Proximity Ligation Assay (PLA)

Cells were seeded onto coverslips (1.5 H thickness, Roth, YX03.1) in 24‐well plates. The following day, cells were incubated with a medium supplemented with EV‐depleted serum for 1 h, and treatments were added to the cells for 2 h. After treatment, cells were fixed with 4% (v/v) formaldehyde and permeabilized with 0.2% (v/v) Triton X‐100 as described above. Duolink PLA reagents (Sigma) were used in the following procedures according to the manufacturer's instructions. Briefly, cells were blocked with Duolink blocking solution for 60 min at 37 °C, and then incubated with primary antibodies at 4 °C for an optimized duration. After washing, cells were incubated with PLUS and MINUS PLA probes for 1 h at 37 °C, and then cells were incubated with ligase for 30 min at 37 °C. After ligation, cells were incubated with polymerase for 100 min at 37 °C. Cells were washed after each step. After final washes, coverslips were mounted using Duolink PLA Mounting Medium with DAPI. Fields were randomly chosen using a systematic sampling method across different regions of each slide. Samples were imaged using Zeiss LSM 900, and the Airyscan detector was used for super‐resolution imaging. Images were processed using ZEN v3.7.

### Electron Microscopy

Quantifoil 2/2 grids were glow discharged in air at 0.2 mbar, 25 mA and 30 s using a Pelco Easyglow. 3 µL sample was added to the grid and blotted away for 3 s using filter paper (Whatman no.4) at 85–95% humidity and room temperature using a Leica EM GP. The grid was subsequently plunged into liquid ethane/propane kept at −196 °C. Grids were transferred to a Talos Arctica (Thermo Fisher Scientific) operated by EPU software in multi‐grid mode. Images were recorded using a K3 direct electron detector (Gatan) in counting mode and ZLP imaging in movie mode at a magnification of 15 000× (corresponding to a pixel size of 0.55 nm at specimen level), a defocus of −5 µm, and a total dose of 35 e A^−2^. Movies were aligned using MotionCor2^[^ ^59^ ^]^ and converted to tiff using EMAN2.^[^ ^60^ ^]^

### RNA Sequencing (RNA‐Seq) and Data Processing

19TT cells were treated with vehicle control or TGF‐β (2.5 ng mL^−1^) for 2 days. The mRNA was isolated and fragmented, followed by cDNA synthesis. The sticky ends of the double‐stranded cDNA were added with A base to the 3′ end by reverse transcription. After amplification by PCR and cyclization, single‐stranded circular DNA (ssCir DNA) was formed as the final library. RNA‐Seq libraries were sequenced on the DNBSEQ platform.

RNA‐Seq files were processed using the opensource BIOWDL RNAseq pipeline v5.0.0 (https://zenodo.org/record/5109461#.Ya2yLFPMJhE) developed at the LUMC. This pipeline performs FASTQ preprocessing (including quality control, quality trimming, and adapter clipping), RNA‐Seq alignment, read quantification, and optionally transcript assembly. FastQC was used for checking raw read QC. Adapter clipping was performed using Cutadapt (v2.10) with default settings and standard illumina universal adapter “AGATCGGAAGAG”. RNA‐Seq reads’ alignment was performed using STAR (v2.7.5a) on GRCh38 human reference genome. The gene read quantification was performed using HTSeq‐count (v0.12.4) with setting “–stranded = reverse”. The gene annotation used for quantification was Ensembl version 105. Using the gene read count matrix, CPM was calculated per sample on all annotated genes for GSEA analysis.

### Single‐Pot, Solid‐Phase‐Enhanced Sample Preparation (SP3) for Mass Spectrometry (MS)

Magnetic beads were brought to room temperature before use. The magnetic bead mixture was prepared by combining 20 µL of Sera‐Mag Speedbeads A (hydrophobic, Thermo Fisher Scientific) and 20 µL of Sera‐Mag Speedbeads B (hydrophilic, Thermo Fisher Scientific) (1:1, v/v). The beads were thoroughly mixed and subjected to sequential washes. First, 160 µL of milli‐Q water (MQ) was used, followed by two additional washes of 200 µL MQ. A magnetic rack was used to settle the beads and to discard the supernatant. After washing, the beads were resuspended in 20 µL MQ to achieve a final working concentration of 100 µg µL^−1^. Beads were stored at 4 °C and resuspended by vortexing prior to use. A 10:1 (w/w) working ratio of SP3 bead mixture/protein as suggested by Hughes et al. was used,^[^ ^61^ ^]^ with a minimum working volume of 10 µL and a minimum bead concentration in working volume of 0.5 µg µL^−1^.

EV lysis and digestion were performed manually as described in Hughes et al.^[^ ^61^ ^]^ For both the con‐EVs and TGF‐β‐EVs, ≈2 × 10^9^ particle equivalents, as measured by MRPS, were used which corresponds to 0.5–12.5 µg, depending on the treatment. In brief, EV lysis was carried out using 1% SDS lysis buffer (100 mm Tris‐HCl, pH 7.6) in a total volume of 16 µL and incubated at 95 °C for 4 min. Subsequently, a one‐step reduction and alkylation was performed with 2 µL of a 0.1 m tris (2‐carboxyethyl) phosphine hydrochloride (TCEP) and 0.4 m chloroacetamide mixture (1:1, v/v), followed by incubation at 95 °C for 5 min. The samples were either directly processed or stored at −20 °C until further use.

For the protein cleanup, 2 µL of the pre‐washed bead mixture was added to the protein lysates along with acetonitrile (ACN) to achieve a final ACN percentage of 50% for optimal protein binding, which was allowed for 18 min at RT. Beads were immobilized on a magnetic rack for 2 min after which the supernatant was discarded. To remove contaminants, beads were washed twice with 200 µL of 80% ethanol and once with 180 µL of 100% ACN off the magnetic rack using pipette mixing, followed by immobilization on the magnetic rack to discard the supernatant. Beads were then resuspended in 15 µL of 100 mm ammonium bicarbonate without pipette mixing to minimize bead clumping. The samples were sonicated for 5 min in a water bath before digestion was performed by adding trypsin (sequencing‐grade trypsin, Promega) with an enzyme/substrate ratio of 1:20, and incubating overnight at 37 °C at 1000 rpm in a thermomixer (Eppendorf ThermoMixer C, Merck). Following digestion, samples were acidified with 5 µL of 5% trifluoroacetic acid. Beads were immobilized on a magnetic rack, and the peptide‐containing supernatant was collected. Samples were lyophilized and stored at −20 °C until use.

### Mass Spectrometry (MS)

Peptides were dissolved in water/formic acid (100/0.1 v/v) and analyzed by on‐line C18 nanoHPLC MS/MS with a system consisting of an Ultimate 3000 nanogradient HPLC system (Thermo Fisher Scientific, Bremen, Germany), and an Exploris480 mass spectrometer (Thermo Fisher Scientific). Samples were injected onto a cartridge precolumn (300 µm × 5 mm, C18 PepMap, 5 µm, 100 A, and eluted via a homemade analytical nano‐HPLC column (50 cm × 75 µm; Reprosil‐Pur C18‐AQ 1.9 µm, 120 A (Dr. Maisch, Ammerbuch, Germany). The gradient was run from 2% to 40% solvent B (20/80/0.1 water/acetonitrile/formic acid (FA) v/v) in 120 min at 250 nL min^−1^. The nano‐HPLC column was drawn to a tip of ≈10 µm and acted as the electrospray needle of the MS source. The mass spectrometer was operated in data‐independent (DIA) MS/MS mode, with a HCD collision energy at 30%. The lock mass of 445.12003 (siloxane) was used. In the master scan the orbitrap resolution was set to 120 000 and the scan range was 350–1100 at a normalized automatic gain control (AGC) of 100. The maximum fill time was set to 20 ms. For targeted MS2 the orbitrap resolution was set to 15 000 with a scan range of 175–2000, at standard AGC. The selected mass windows were ≈11 Th wide, with ≈9 points acquired across the peaks.

Data were processed using Spectronaut 18 (Biognosys AG) with default settings. Carbamidomethyl was set as a fixed modification, and methionine oxidation was set as a dynamic modification. Missing values were not imputed, and the proteins with at least two valid values in at least one group remained. Proteins with an absolute fold change >1.5 and *p*‐value < 0.05 between the two groups were recognized as differentially expressed proteins.

### Statistics

All statistical analyses were performed using GraphPad Prism 10. Unless otherwise indicated, the results are shown as mean ± standard deviation (SD). Two‐sided statistical tests were performed in all statistical analyses. Differences were considered statistically significant at *p* < 0.05.

## Conflict of Interest

The authors declare no conflict of interest.

## Author Contributions

C.L. and P.T.D. designed the study. C.L., Z.L., S.R., and R.I.K. performed experiments and C.L., Z.L., S.R., P.V.V., R.I.K., and H.M. analyzed the data. C.L. wrote the manuscript and P.T.D., A.E.M., S.R., P.V.V., R.I.K. and H.M. revised the manuscript. All authors approved the final version of the paper.

## Supporting information



Supporting Information

Supplemental Video 1

Supplemental Video 2

Supplemental Video 3

## Data Availability

The RNA‐seq data have been deposited in the European Genome‐phenome Archive (EGA) under the accession number EGAD50000001350. The mass spectrometry proteomics data have been deposited to the ProteomeXchange Consortium via the PRIDE partner repository with the dataset identifier PXD061656. All relevant data are available from the authors.
