# Supplementary material for: Surface‐Associated Proteins on Extracellular Vesicles Remodel the Tumor Microenvironment by Potentiating TGF‐β Signaling in a Contact‐Dependent Manner
Source: Adv Sci (Weinh). 2025 Nov 30;13(8):e13286. doi: 10.1002/advs.202513286 (PMC12884746; doi:10.1002/advs.202513286)
Supplement: Supplementary file 1 — Supporting Information [file ADVS-13-e13286-s001.docx]

Supplementary Information

**Surface-associated proteins on extracellular vesicles remodel the tumor microenvironment by potentiating TGF-β signaling in a contact-dependent manner**

Chao Li^1^, Agustin Enciso-Martinez^1,2,3^, Lizhe Zhu^1^, Sarah A. Rotman^4^, Peter A. van Veelen^4^, Roman I. Koning^5^, Hailiang Mei^6^, Peter ten Dijke^1*^

^1^ Oncode Institute and Department of Cell and Chemical Biology, Leiden University Medical Center, Leiden, the Netherlands.

^2^ Amsterdam UMC, University of Amsterdam, Biomedical Engineering & Physics; Amsterdam Cardiovascular Sciences, Cancer Center Amsterdam, Meibergdreef 9, Amsterdam, the Netherlands

^3^ Amsterdam UMC, University of Amsterdam, Laboratory of Experimental Clinical Chemistry; Laboratory Specialized Diagnostics & Research, Department of Laboratory Medicine, Meibergdreef 9, Amsterdam, the Netherlands

^4^ Center for Proteomics and Metabolomics, Leiden University Medical Center, Leiden, the Netherlands.

^5^ Electron Microscopy Facility, Department of Cell and Chemical Biology, Leiden University Medical Center, Leiden, the Netherlands.

^6^ Department of Biomedical Data Sciences, Sequencing Analysis Support Core, Leiden University Medical Center, Leiden, the Netherlands.

*Correspondence to Peter ten Dijke ([p.ten_dijke@lumc.nl](mailto:p.ten_dijke@lumc.nl))


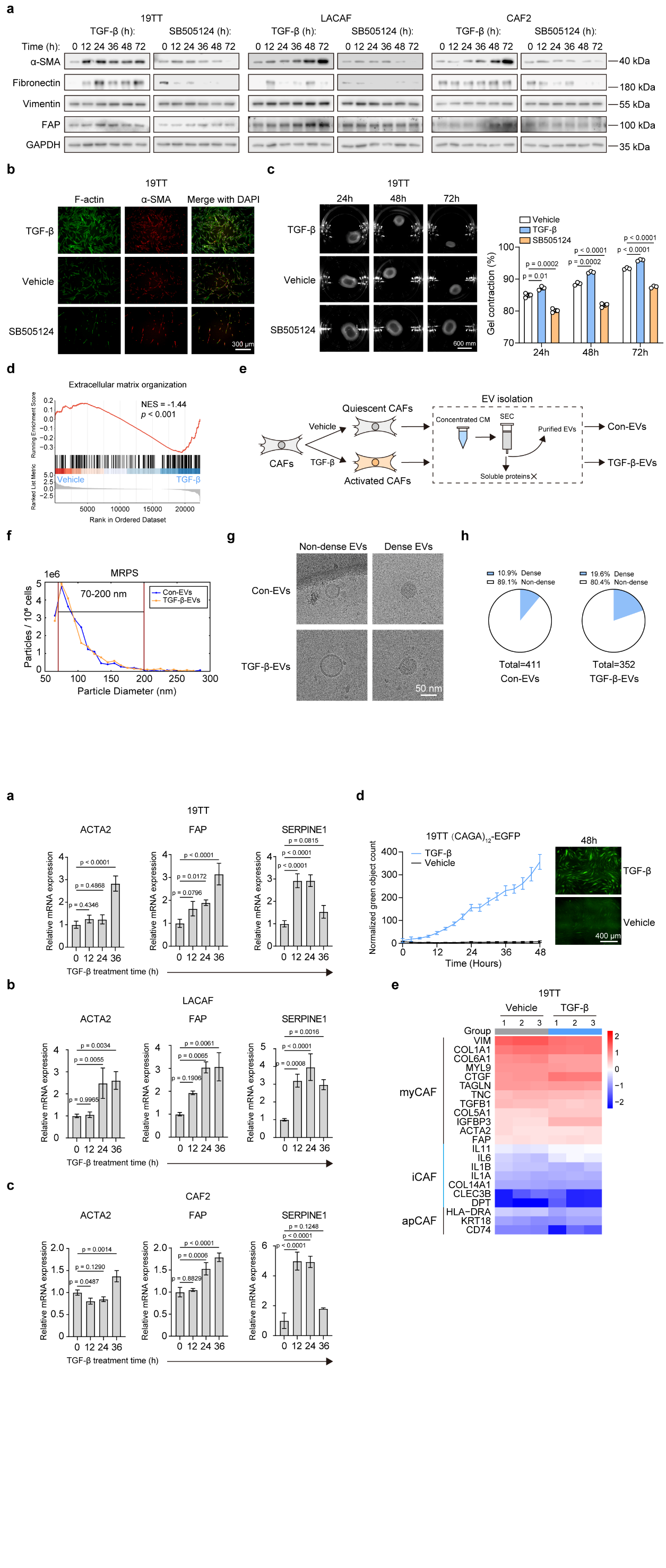


**Supplementary Figure 1. TGF-β promotes CAF activation.** (a-c) Real-time PCR analysis of the mRNA expression of *ACTA2*, *FAP*, and *SERPINE1* in response to TGF-β treatment in three different CAF cell lines, i.e. 19TT, LACAF and CAF2. CAFs were treated with TGF-β for 0, 12, 24, and 36 hours. Means ± SD, n = 3 biological replicates, one-way ANOVA with Dunnett’s test. Representative of n = 2 experiments. (d) Real-time imaging of (CAGA)_12_-SMAD3 transcriptional EGFP reporter activity in 19TT CAFs in response to vehicle control or TGF-β treatment. The quantification of normalized green object counts is shown in the left panel. Means ± SD. Representative images after 48 hours of treatment are shown in the right panel. The scale bar represents 400 μm. Representative of n = 3 experiments. (e) The mRNA expression of typical markers of myofibroblastic CAFs (myCAF), inflammatory CAFs (iCAFs), and antigen-presenting CAFs (apCAFs) from RNA-seq transcriptional profiling of 19TT CAFs treated with vehicle control or TGF-β. The expression values are z-score transformed.


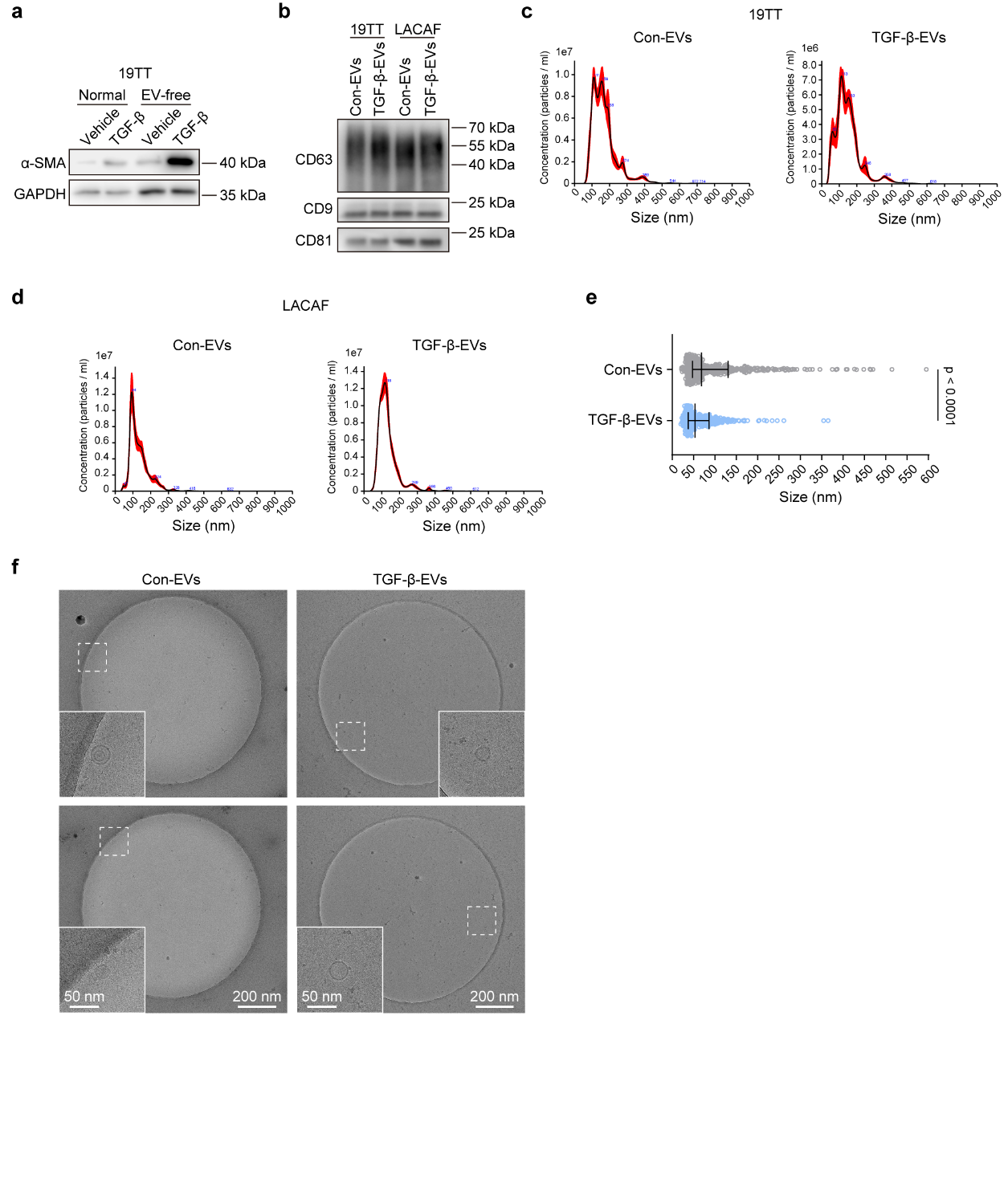


**Supplementary Figure 2. Characterization of CAF-derived EVs.** (a) Western blot analysis showing the effect of TGF-β on CAF activation in a medium supplemented with normal serum or EV-depleted serum. Representative of n = 3 experiments. (b) Western blot analysis of tetraspanin markers CD9, CD63, and CD81 of con-EVs and TGF-β-EVs derived from 19TT and LACAF cells based on approximately equal particle numbers. Representative of n = 3 experiments. (c, d) Nanoparticle tracking analysis (NTA) of con-EVs and TGF-β-EVs derived from 19TT and LACAF cells. Representative of n > 3 experiments. (e) Size distribution of con-EVs and TGF-β-EVs derived from 19TT cells characterized by cryo-EM. Medians with interquartile ranges are shown in the graph. Unpaired student’s t-test. (f) Representative cryo-EM images of con-EVs and TGF-β-EVs, from >200 and >500 images, respectively. Pure EV preparations, free of visible protein or EV aggregates, were prepared from 19TT cells.


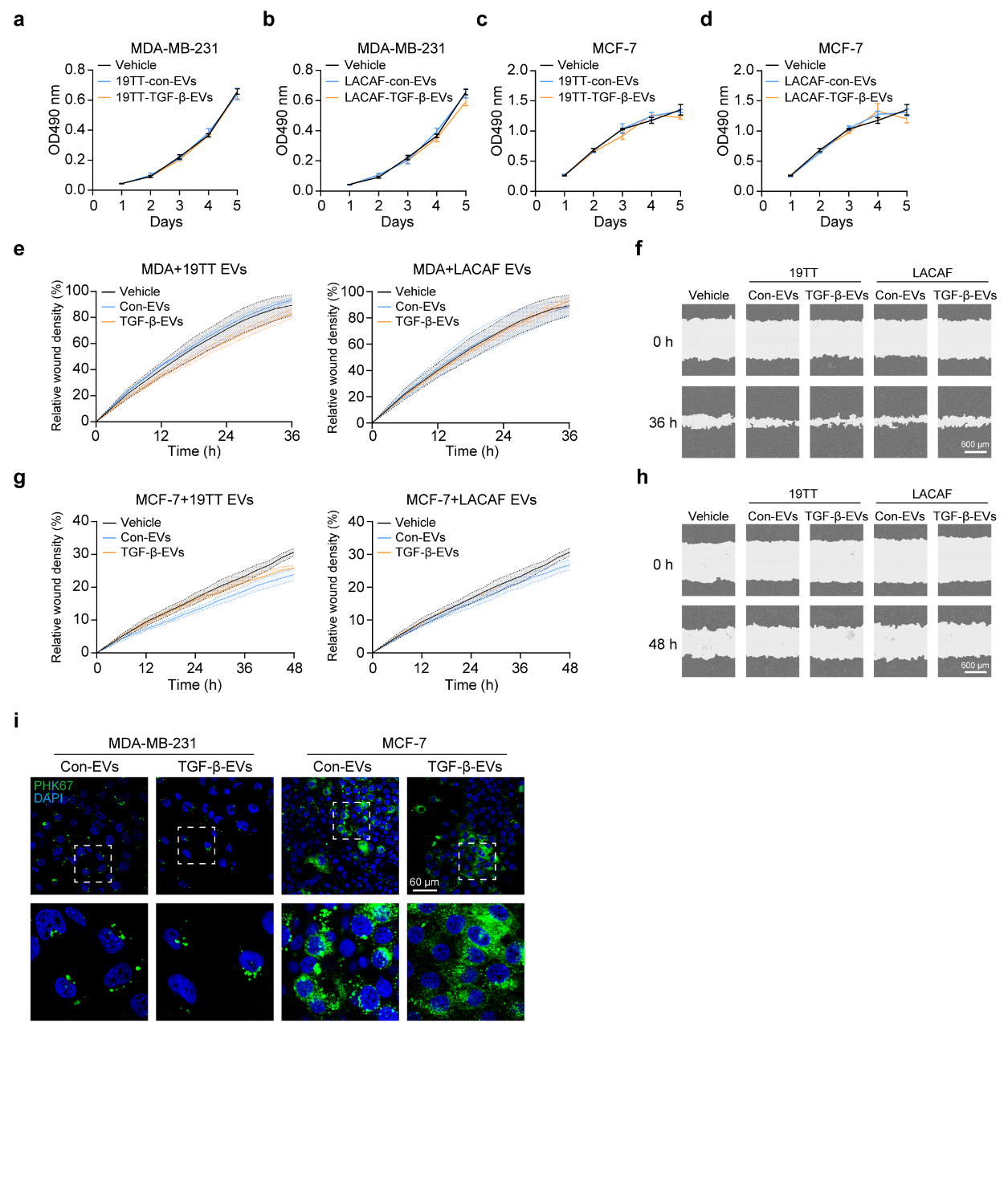


**Supplementary Figure 3. The effect of CAF-derived EVs on cancer cells.** (a-d) The proliferation of MDA-MB-231 and MCF-7 cells treated with con-EVs or TGF-β-EVs derived from 19TT and LACAF cells. The cell proliferation was measured by MTS assay and recorded for 5 days. Means ± SD, n = 3 biological replicates. Representative of n = 3 experiments. (e-h) Wound healing assay showing the effect of EVs derived from 19TT and LACAF cells on the migration of MDA-MB-231 and MCF-7 cells. The same vehicle controls were used for 19TT and LACAF cell-derived EV treatment groups. Means ± SD, n = 3 biological replicates. Representative wound images of MDA-MB-231 and MCF-7 cells were shown in (f) and (h), respectively. The scale bar represents 600 μm. Representative of n = 3 experiments. (i) Immunofluorescence analysis of the uptake of PHK67-labeled EVs derived from 19TT cells by MDA-MB-231 and MCF-7 cells after 4 hours of incubation. The magnified regions are shown below. The scale bar represents 60 μm. Representative of n = 2 experiments.


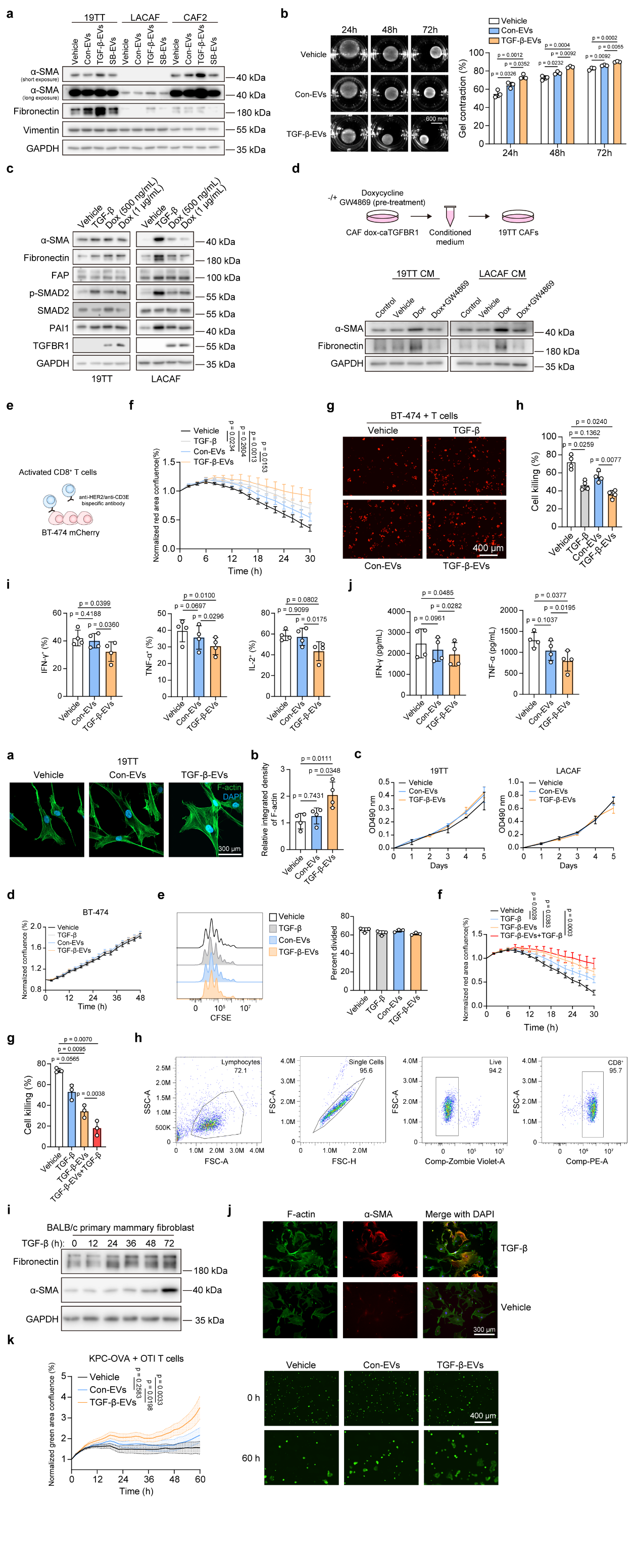


**Supplementary Figure 4. The function of CAF-derived EVs.** (a) Analysis of F-actin of 19TT cells by phalloidin staining treated with vehicle control, or EVs derived from 19TT cells. The scale bar represents 300 μm. Representative of n = 3 experiments. (b) Quantification of relative integrated density of F-actin from (a). Representative of n = 3 experiments. (c) Proliferation of 19TT and LACAF cells treated with vehicle control or EVs derived from 19TT cells, measured by MTS assay. Means ± SD, n = 3 biological replicates. Representative of n = 3 experiments. (d) Proliferation of BT-474 cells treated with vehicle control, TGF-β, or EVs derived from 19TT cells tracked by Incucyte live-cell analysis system. Means ± SD, n = 3 biological replicates. Representative of n = 3 experiments. (e) Proliferation of carboxyfluorescein succinimidyl ester (CFSE)-stained CD8⁺ T cells measured by flow cytometry. BT-474 mCherry cells were seeded in 48-well plates at a density of 15,000 cells per well. CFSE-stained activated CD8⁺ T cells were added at an effector-to-target (E:T) ratio of 4:1 in the presence of an anti-HER2/anti-CD3E bispecific antibody (100 ng/mL) and indicated treatments. CFSE fluorescence from CD8⁺ T cells was measured after 2 days of co-culture. Left: Representative of n = 3 donors. Right: Quantification of the percentage of divided cells. One-way repeated measures ANOVA with Tukey’s test with n = 3 donors. (f) Real-time tracking of BT-474 mCherry cells co-cultured with activated CD8^+^ T cells with indicated treatments. Means ± SD, n = 3 biological replicates, one-way ANOVA with Tukey’s test. Representative of n = 3 donors. (g) Quantification of the cytotoxicity of CD8^+^ T cells in the co-culture. The percentages of cancer cells killed by CD8^+^ T cells were calculated using the cancer cell monoculture as a control. One-way repeated measures ANOVA with Tukey’s test with n = 3 donors. (h) Gating strategy used to identify single, live human primary CD8^+^ T cells in flow cytometry analysis. (i) Western blot analysis of the effect of TGF-β on the activation of primary mammary fibroblasts from BALB/c mice. Representative of n = 2 experiments. (j) Phalloidin and α-SMA fluorescent staining in primary mammary fibroblasts from BALB/c mice with vehicle control or TGF-β treatment. The scale bar represents 300 μm. Representative of n = 2 experiments. (k) Real-time tracking of mouse KPC-OVA pancreatic cancer cells co-cultured with activated mouse OT-I CD8^+^ T cells with indicated treatments. OT-I CD8^+^ T cells were seeded in the plates with an effector-to-target ratio of 1:2. Representative images of different conditions at 0 hour and 60 hours were shown, and the scale bar represents 400 μm. Means ± SD, n = 3 biological replicates, one-way ANOVA with Tukey’s test. Representative of n = 3 experiments.


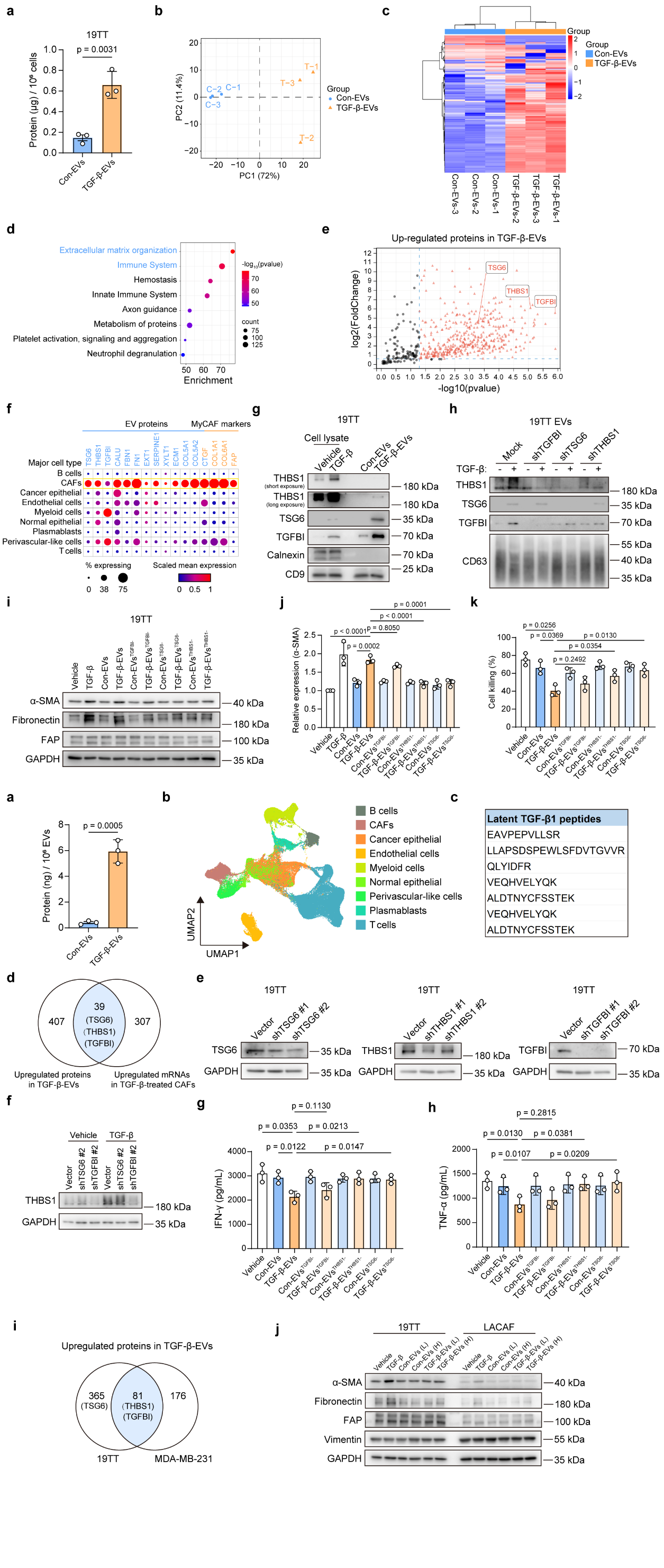


**Supplementary Figure 5. Functions of most potently upregulated proteins in TGF-β-EVs as compared to con-EVs identified by proteomics.** (a) Protein quantification of 19TT-derived EVs normalized to an equal particle number of EVs measured by microfluidic resistive pulse sensing (MRPS). Means ± SD, n = 3 biological replicates, unpaired student’s t-test. Representative of n = 3 experiments. (b) UMAP visualization showing cell type clusters derived from a single-cell RNA sequencing dataset (GSE176078) from Single cell PORTAL. (c) Peptides corresponding to latent TGF-β1 (and not TGF-β3) identified by proteomic analysis. (d) Venn diagram showing the overlap between upregulated proteins in TGF-β-EVs and genes upregulated in mRNA expression upon TGF-β treatment of 19TT cells. (e) Validation of shRNA-mediated knockdown of TSG6, THBS1, and TGFBI in 19TT cells. Representative of n = 2 experiments. (f) Effect of TSG6 and TGFBI depletion on the protein expression of THBS1 in 19TT cells. Representative of n = 3 experiments. (g, h) Secreted IFN-γ and TNF-α from activated CD8^+^ T cells measured by ELISA assay. CD8^+^ T cells were incubated with indicated treatments for 2 days. One-way repeated measures ANOVA with Tukey’s test with n = 3 donors. (i) Venn diagram showing the overlap and differences in upregulated proteins in TGF-β-EVs derived from 19TT and MDA-MB-231 cells. (j) Western blot analysis showing the effect of MDA-MB-231 cell-derived EVs on the activation of 19TT and LACAF cells. L: Low EV concentration (1×10^8^ particles/mL). H: High EV concentration (4×10^8^ particles/mL). Representative of n = 3 experiments.


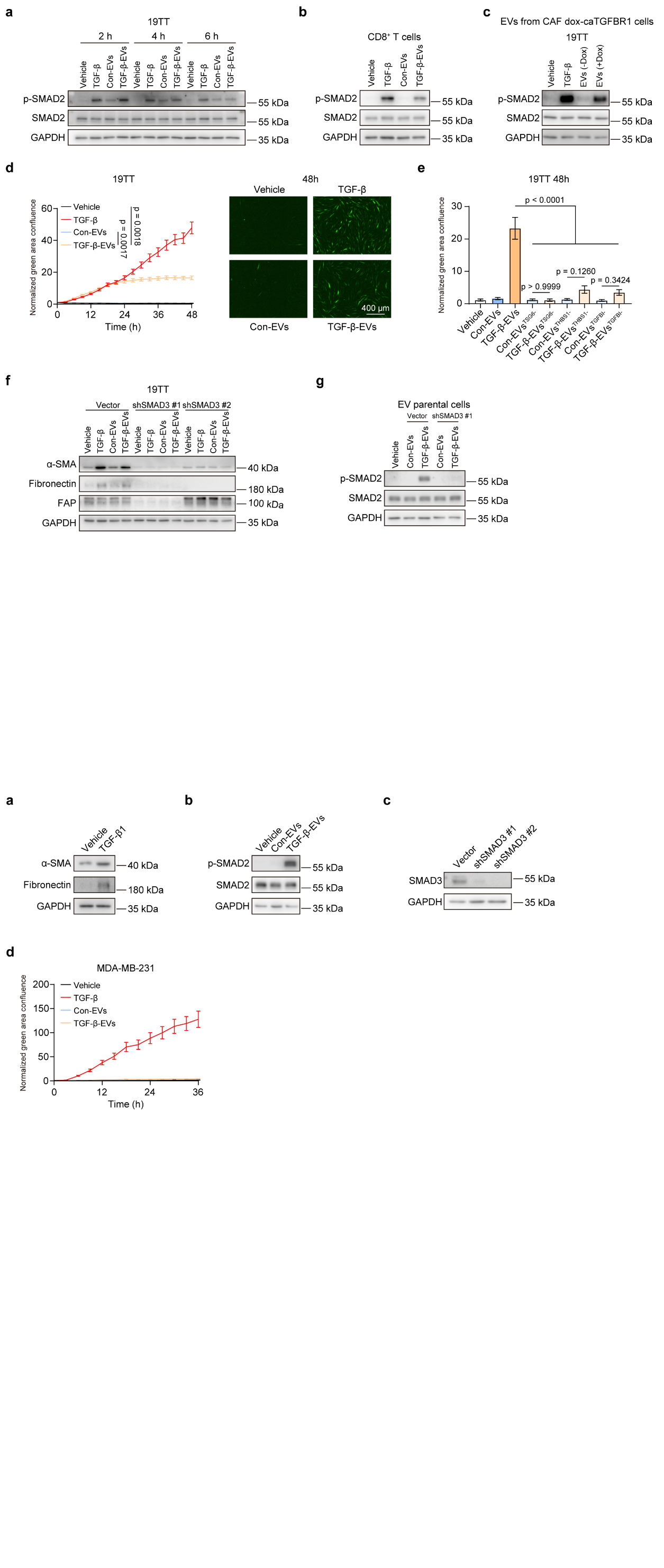


**Supplementary Figure 6. Effect of EVs on the activation of TGF-β signaling.** (a) Effect of TGF-β1 (2.5 ng/mL) on CAF activation. 19TT CAFs were treated with TGF-β1 for 2 days. Representative of n = 2 experiments. (b) Effect of EVs derived from TGF-β1-activated 19TT CAFs on the induction of TGF-β signaling in 19TT CAFs. 19TT CAFs were treated with EVs for 2 hours. Representative of n = 2 experiments. (c) Validation of shRNA-mediated knockdown of SMAD3 in 19TT cells. #1 and #2 are two independent shRNAs; GAPDH, loading control Representative of n = 2 experiments. (d) Real-time imaging of (CAGA)_12_-EGFP reporter-stably-expressing MDA-MB-231 cells incubated with vehicle, TGF-β, and 19TT cell-derived EVs. The quantification of normalized green area confluence is shown. Means ± SD. Representative of n = 3 experiments.


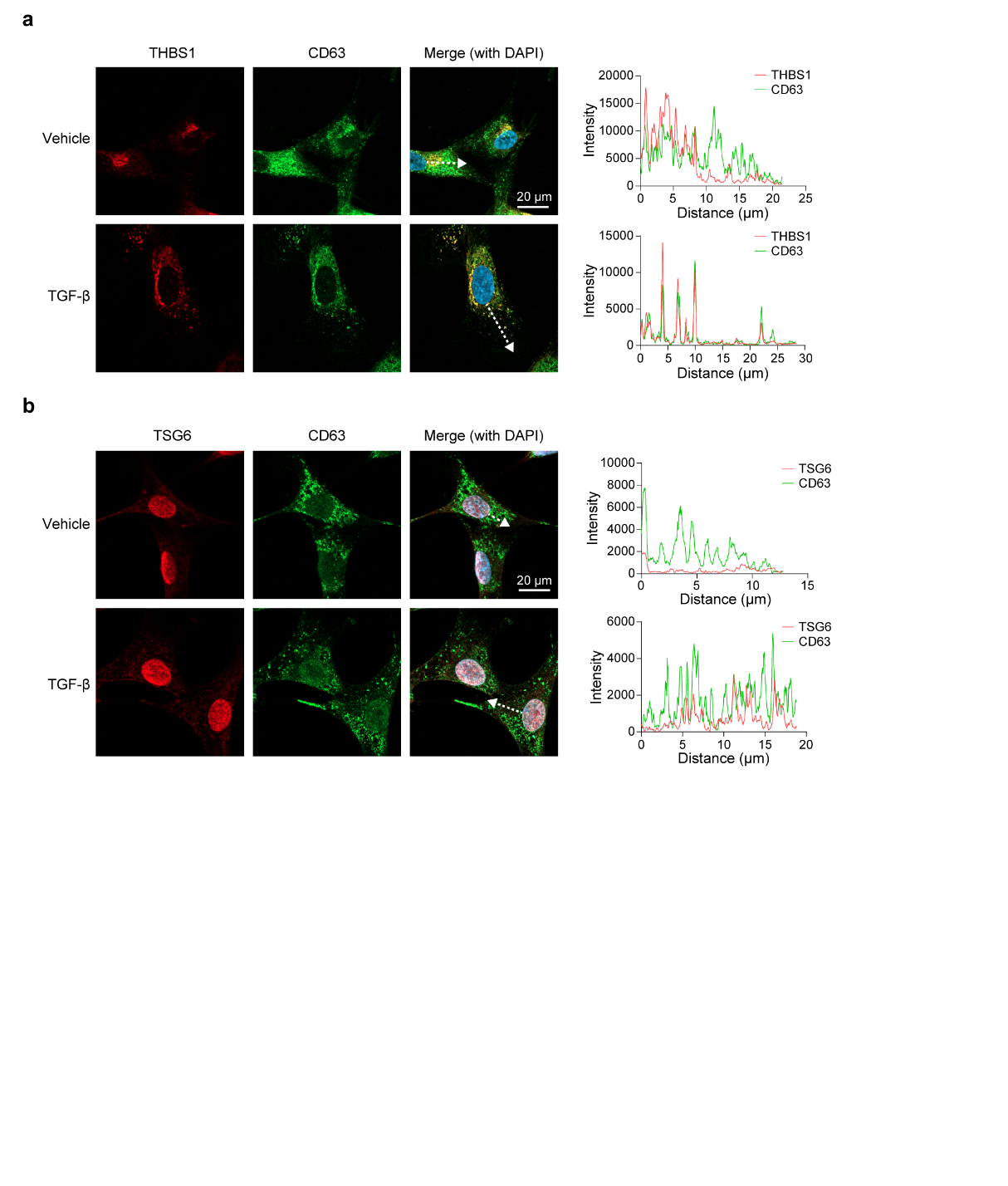


**Supplementary Figure 7. Localization of THBS1 and TSG6 in CAFs.** (a, b) Immunofluorescence analysis of the localization of THBS1, TSG6, and CD63 in 19TT cells. Cells were treated with vehicle control or TGF-β for 2 days. The intensity distributions of THBS1, TSG6, and CD63 from perinuclear to cell periphery (along the white arrow) are shown in the right panels. The scale bar represents 20 μm. Representative of n = 3 experiments.


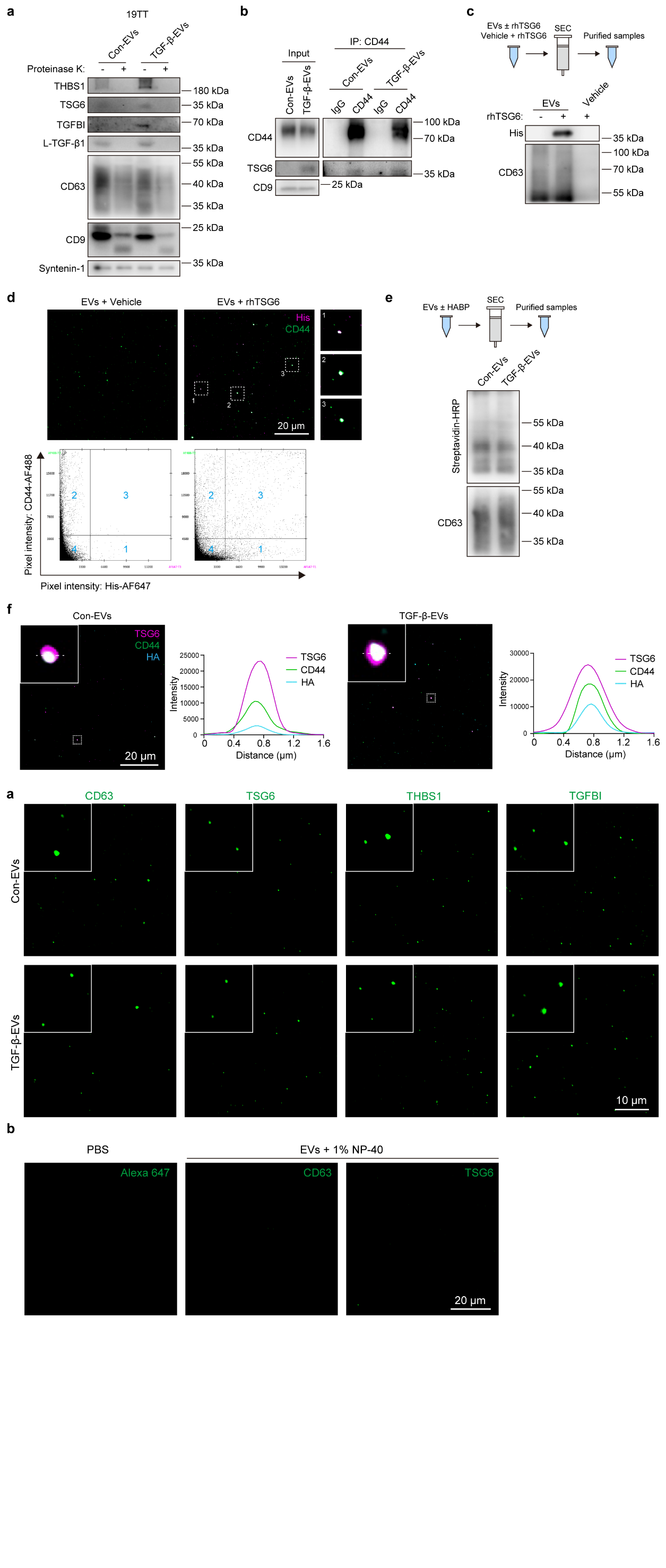


**Supplementary Figure 8. Characterization of surface-associated proteins on CAF derived-EVs.** (a) Super-resolution microscopy (SRM) imaging of con-EVs and TGF-β-EVs with immunofluorescence-labeling of CD63, TSG6, THBS1, and TGFBI on the EV surface. The scale bar represents 10 μm. The magnified regions are shown in the upper left corner. Representative of n = 3 experiments. (b) PBS control and 1% NP-40 detergent controls related to (a).


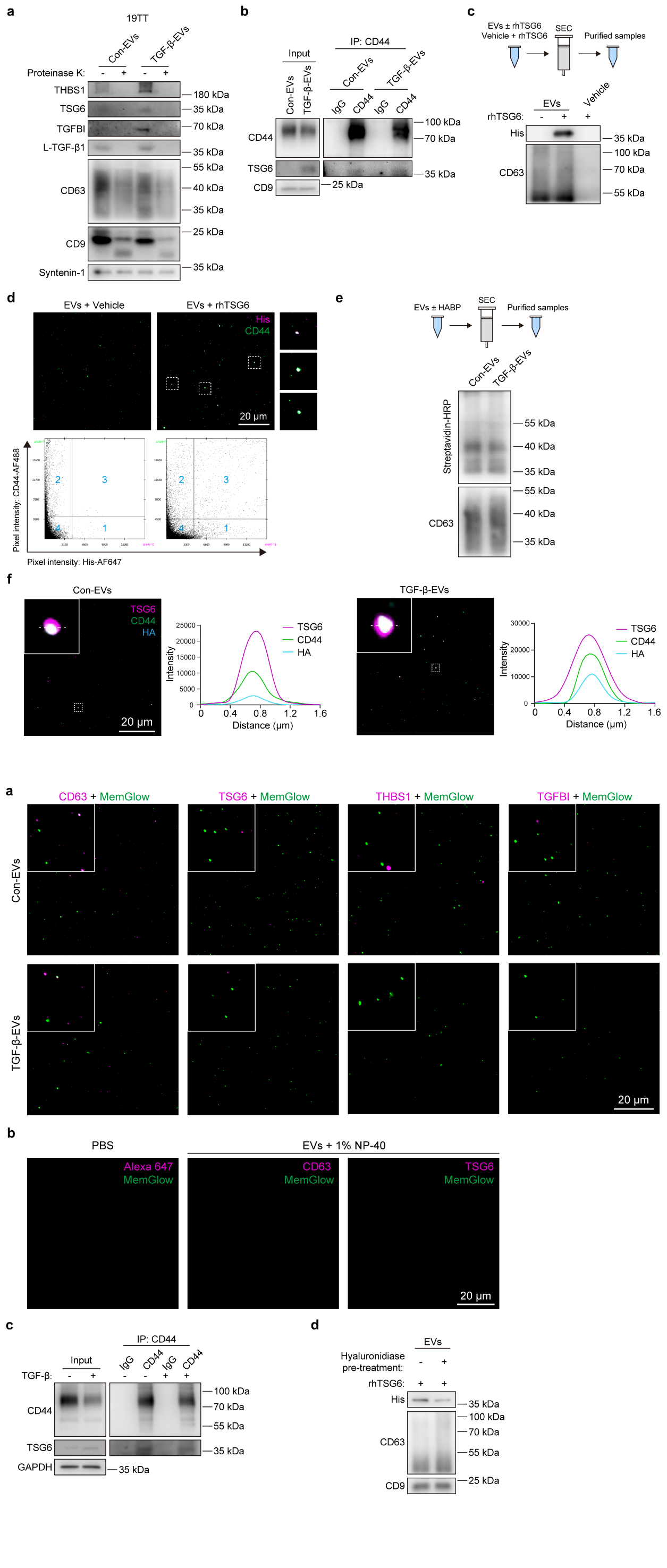


**Supplementary Figure 9. Characterization of surface-associated proteins on CAF derived-EVs.** (a) Super-resolution microscopy (SRM) imaging of immunofluorescence-labeled CD63, TSG6, THBS1, TGFBI, and MemGlow on the surface of proteinase K-treated con-EVs and TGF-β-EVs. The scale bar represents 20 μm. Representative of n = 3 experiments. (b) PBS control and 1% NP-40 detergent controls related to (a). The scale bar represents 20 μm. The magnified regions are shown in the upper left corner. Representative of n = 3 experiments. (c) Co-immunoprecipitation assay demonstrating the interaction between TSG6 and CD44 in 19TT cells. Representative of n = 2 experiments. (d) Western blot analysis of the binding of recombinant human TSG6 (rhTSG6) to 19TT cell-derived EVs with or without hyaluronidase pre-treatment. EVs were purified using size exclusion chromatography (SEC) after hyaluronidase pre-treatment and rhTSG6 incubation. Representative of n = 3 experiments.


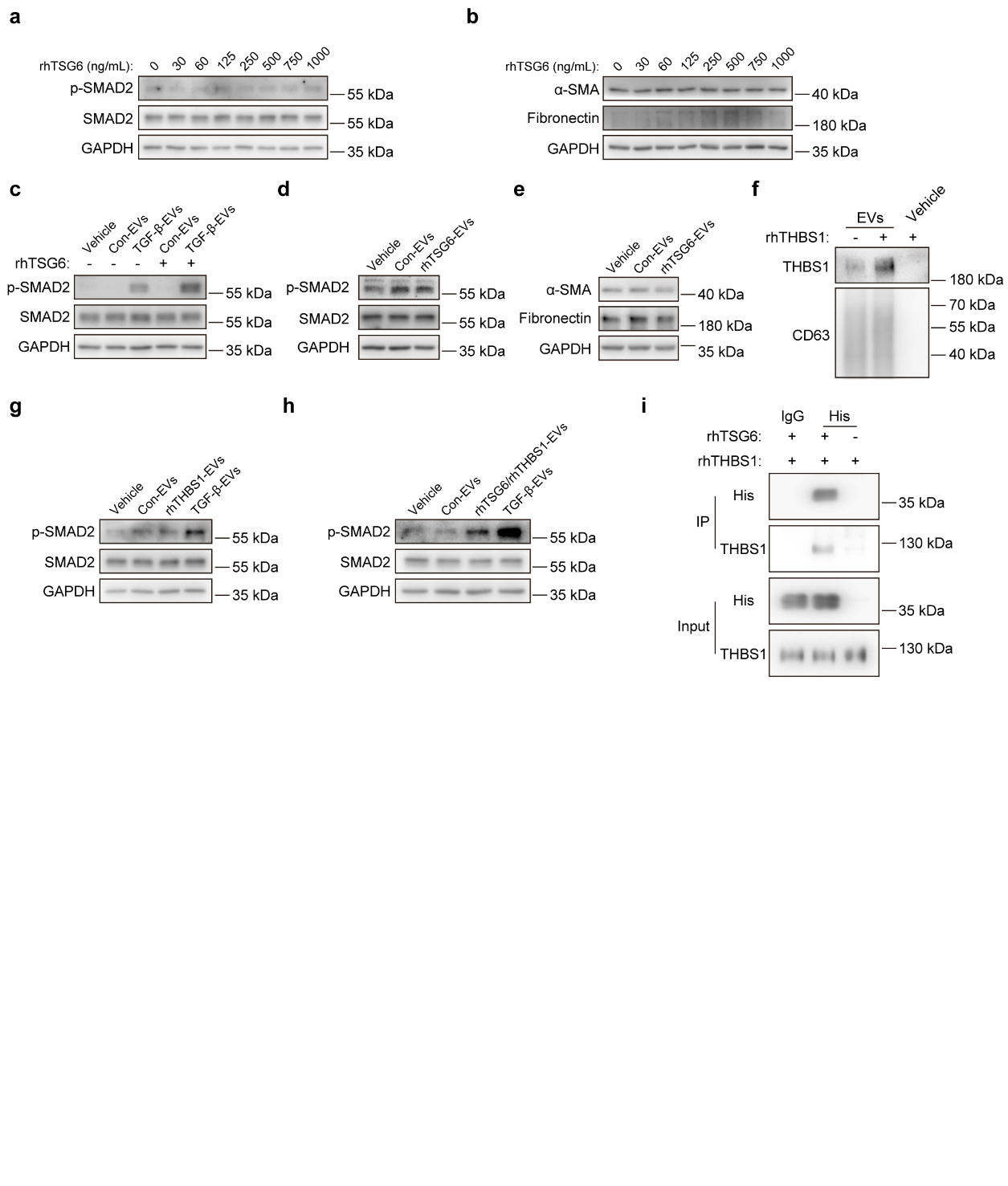


**Supplementary Figure 10. The role of TSG6 and THBS1 in CAF-derived EV-mediated TGF-β signaling.** (a) Western blot analysis showing the effect of soluble recombinant human TSG6 (rhTSG6) protein on TGF-β signaling. Representative of n = 3 experiments. (b) CAF activation in response to soluble rhTSG6 protein. Representative of n = 3 experiments. (c) Effect of rhTSG6 (200 ng/mL) in 19TT CAF-derived EVs on the induction of TGF-β signaling. 19TT CAFs were treated with indicated treatments for 2 hours. Representative of n = 3 experiments. (d) Effect of rhTSG6-coated EVs on TGF-β signaling. Representative of n = 3 experiments. (e) Effect of rhTSG6-coated EVs on CAF activation. Representative of n = 3 experiments. (f) Analysis of the interaction of recombinant human THBS1 (rhTHBS1) with 19TT cell-derived EVs. Representative of n = 2 experiments. (g) The effect of rhTHBS1-coated EVs on TGF-β signaling. Representative of n = 3 experiments. (h) Effect of 19TT cell-derived EVs coated with both rhTSG6 and rhTHBS1 on TGF-β signaling. Representative of n = 3 experiments. (i) Co-immunoprecipitation assay detecting the interaction between His-rhTSG6 and rhTHBS1. Representative of n = 3 experiments.


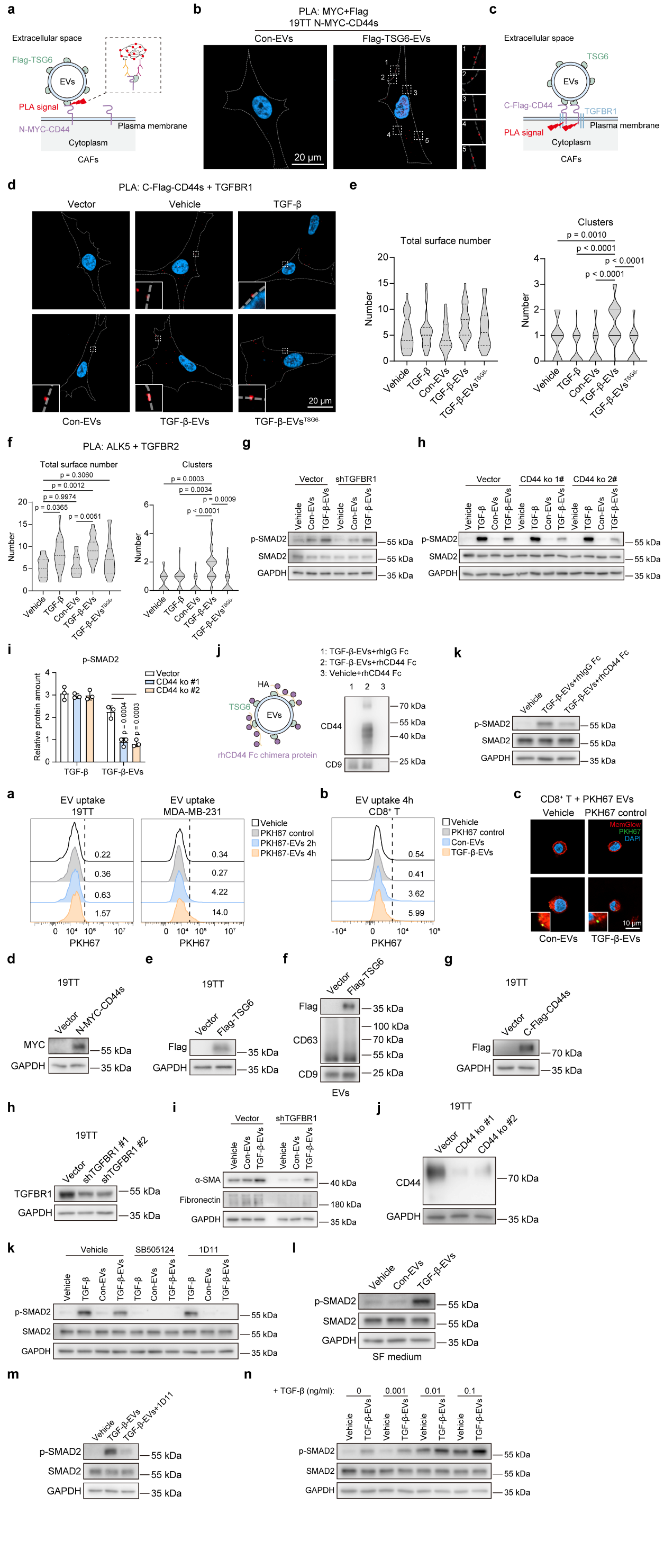


**Supplementary Figure 11. TGF-β-EVs potentiate TGF-β signaling in a contact-dependent manner.** (a) Flow cytometry analysis of the uptake of PKH67-labeled 19TT-derived EVs in 19TT and MDA-MB-231 cells. Representative of n = 3 experiments. (b) Flow cytometry analysis of the uptake of PKH67-labeled 19TT-derived EVs in activated CD8^+^ T cells. Representative of n = 3 experiments. (c) Confocal microscopy imaging of the uptake of PKH67-labeled EVs in activated CD8^+^ T cells. The cell membrane was labeled with MemGlow 590. Representative of n = 2 experiments. The scale bar represents 10 μm. (d, e) Validation of the overexpression of N-MYC-CD44s and Flag-TSG6 in 19TT cells. Representative of n = 2 experiments. (f) Validation of the expression of Flag-TSG6 in EVs derived from Flag-TSG6 overexpressing 19TT cells. Representative of n = 2 experiments. (g) Validation of the overexpression of C-Flag-CD44s in 19TT cells. Representative of n = 2 experiments. (h) Validation of shRNA-mediated knockdown of TGFBR1 in 19TT cells. Representative of n = 2 experiments. (i) Western blot analysis showing the effect of shRNA-mediated TGFBR1 knockdown on EV-mediated 19TT CAF activation. Representative of n = 3 experiments. (j) Validation of the (near to complete) depletion of CD44 in CRISPR-Cas9-mediated CD44 knockout 19TT cells. Representative of n = 2 experiments. (k) Western blot analysis showing the effect of pan-TGF-β neutralizing antibody 1D11 and selective small molecule TGFBR1 inhibitor SB505124 on EV-mediated TGF-β signaling in 19TT cells. 1D11 and SB505124 was preincubated with treatments in the medium for 30 minutes before being adding to the cells. 19TT cells were incubated with indicated treatments for 2 hours. Representative of n = 3 experiments. (l) Western blot analysis showing the effect of 19TT cell-derived EVs on TGF-β signaling in 19TT cells. 19TT cells were incubated with EVs in serum free medium for 2 hours. Representative of n = 3 experiments. (m) Western blot analysis showing the effect of coating TGF-β-EVs with the pan-TGF-β-neutralizing antibody 1D11 on their ability to induce TGF-β signaling. 19TT cells were treated with EVs for 2 hours. Representative of n = 3 experiments. (n) Western blot analysis showing the effect of TGF-β-EVs on TGF-β signaling with various low concentrations of TGF-β. 19TT cells were treated with EVs for 2 hours. Representative of n = 3 experiments.


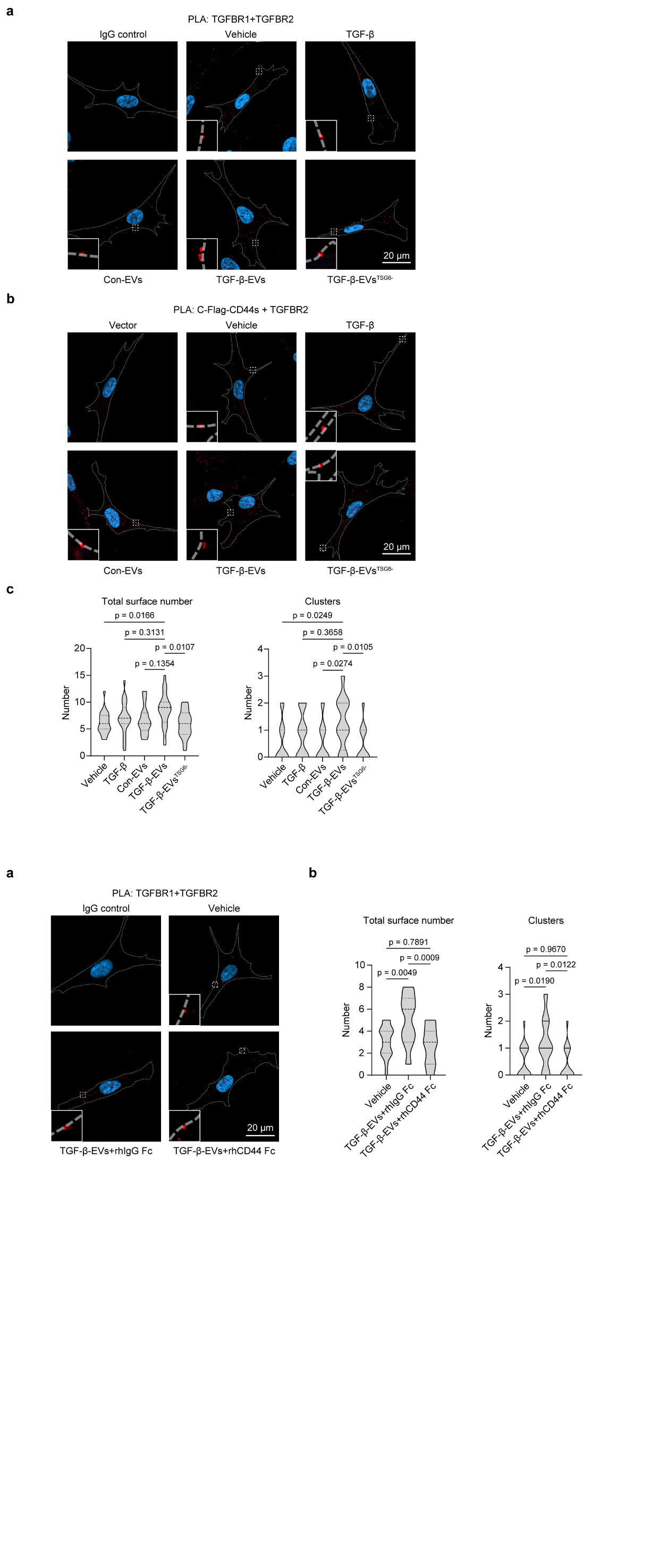


**Supplementary Figure 12. TGF-β-EVs induce receptor clustering of CD44s and TGF-β receptors on the cell membrane.** (a) Super-resolution microscopy (SRM) imaging of proximity ligation assay (PLA) targeting TGFBR1 and TGFBR2 in 19TT cells. Cells were incubated with indicated treatments for 2 hours. The scale bar represents 20 μm. Representative of n = 3 experiments. (b) SRM imaging of PLA targeting C-Flag-CD44s and TGFBR2 in 19TT cells. Cells were incubated with indicated treatments for 2 hours. The scale bar represents 20 μm. Representative of n = 3 experiments. (c) Quantification of total PLA signals and clustering events on the cell surface per cell from (b). More than 20 cells per condition were used for quantification. Representative of n = 3 experiments.


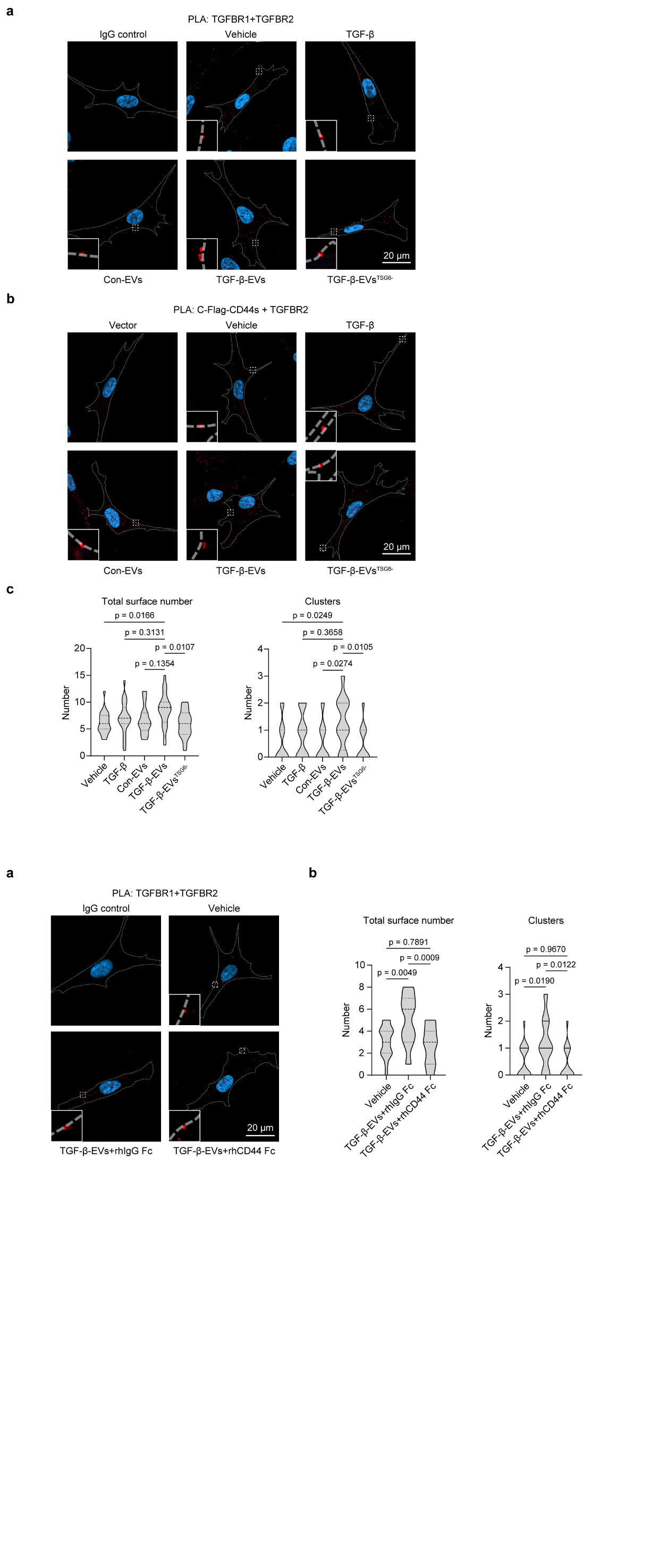


**Supplementary Figure 13. The role of CD44 binding sites on TGF-β-EVs in inducing receptor clustering of TGF-β receptors.** (a) Super-resolution microscopy (SRM) imaging of proximity ligation assay (PLA) targeting TGFBR1 and TGFBR2 in 19TT cells. TGF-β-EVs were pre-coated with rhIgG-Fc or rhCD44-Fc chimera protein. Cells were incubated with indicated treatments for 2 hours. The scale bar represents 20 μm. Representative of n = 3 experiments. (b) Quantification of total PLA signals and clustering events on the cell surface per cell from (a). More than 20 cells per condition were used for quantification. Representative of n = 3 experiments.

**Supplementary Table 1. The list of recombinant proteins**

| Recombinant protein | Species | Company | Cat # |
| --- | --- | --- | --- |
| TSG6 | Human | R&D systems | 2104-TS |
| THBS1 | Human | Sigma | ECM-002 |
| CD44 Fc Chimera | Human | R&D systems | 3660-CD |
| IgG1 Fc | Human | R&D systems | 110-HG |

**Supplementary Table 2. The list of shRNA constructs used in this study from Mission shRNA library of Sigma-Aldrich**

| Gene | Target region | Species | TRC Id |
| --- | --- | --- | --- |
| TSG6 (#1) | CDS | Human | TRCN0000115988 |
| TSG6 (#2) | CDS | Human | TRCN0000115989 |
| THBS1 (#1) | CDS | Human | TRCN0000226402 |
| THBS1 (#2) | CDS | Human | TRCN0000226403 |
| TGFBI (#1) | CDS | Human | TRCN0000062174 |
| TGFBI (#2) | CDS | Human | TRCN0000062176 |
| SMAD3 (#1) | CDS | Human | TRCN0000020009 |
| SMAD3 (#2) | CDS | Human | TRCN0000020010 |
| TGFBR1 (#1) | 3′ UTR | Human | TRCN0000039773 |
| TGFBR1 (#2) | CDS | Human | TRCN0000039774 |

**Supplementary Table 3. The list of antibodies for western blot**

| Antibody | Clone | Company | Cat # | Dilution |
| --- | --- | --- | --- | --- |
| α-SMA | 1A4 | Sigma | A2547 | 1:1000 |
| Fibronectin | FN-15 | Sigma | F7387 | 1:1000 |
| Vimentin | D21H3 | Cell signaling | 5741 | 1:1000 |
| FAP | 1E5 | Sigma | WH0002191M1 | 1:1000 |
| GAPDH | 6C5 | Millipore | MAB374 | 1:2000 |
| CD63 | MX-49.129.5 | Santa Cruz | Sc-5275 | 1:1000 |
| CD9 | HI9a | Biolegend | 312102 | 1:1000 |
| CD81 | B-11 | Santa Cruz | Sc-166029 | 1:1000 |
| SMAD2 | EP784Y | Epitomics | 1736-1 | 1:1000 |
| PAI1 | 41/PAI-1 | BD Biosciences | 612025 | 1:1000 |
| TGFBR1 | V-22 | Santa Cruz | sc-398 | 1:1000 |
| THBS1 | A6.1 | Thermofisher | MA5-13398 | 1:1000 |
| TSG6 | D-4 | Santa Cruz | sc-398307 | 1:1000 |
| TGFBI | D31B8 | Cell signaling | 5601 | 1:1000 |
| Calnexin | Polyclonal | Abcam | Ab22595 | 1:1000 |
| SMAD3 | EP568Y | Epitomics | 1735-1 | 1:1000 |
| TGF-β | 1D11 | R&D systems | MAB1835 | 1:1000 |
| Syntenin-1 | E2I9L | Cell signaling | 27964 | 1:1000 |
| CD44 | EPR18668 | Abcam | Ab189524 | 1:1000 |
| His | HIS.H8 | Thermofisher | MA1-21315 | 1:1000 |
| Streptavidin-HRP |  | Cell signaling | 3999s | 1:2000 |
| MYC | 9E10 | Sigma | M4439 | 1:1000 |
| Flag | M2 | Sigma | F1804 | 1:1000 |
| Flotillin-1 | D2V7J | Cell signaling | 18634 | 1:1000 |
| TfR | H68.4 | Thermofisher | 13-6800 | 1:1000 |
| EEA1 | 1G11 | Thermofisher | 14-9114-80 | 1:1000 |

**Supplementary Table 4. The list of primers for real-time PCR**

| Primer: | Target species | Sequence (5′ - 3′): |
| --- | --- | --- |
| GAPDH-F | Human | TGCACCACCAACTGCTTAGC |
| GAPDH-R | Human | GGCATGGACTGTGGTCATGAG |
| α-SMA-F | Human | CTGTTCCAGCCATCCTTCATC |
| α-SMA-R | Human | CCGTGATCTCCTTCTGCATT |
| FAP-F | Human | CAATGTGGTACTCTGACCAGAACC |
| FAP-R | Human | TCTGATACAGGCTTGCATCTGC |
| SERPIN1-F | Human | CACAAATCAGACGGCAGCACT |
| SERPIN1-R | Human | CATCGGGCGTGGTGAACTC |

**Supplementary Table 5. The list of antibodies for flow cytometry**

| Antibody | Fluorochrome | Clone | Company | Cat # | Dilution |
| --- | --- | --- | --- | --- | --- |
| IFN-γ | FITC | 4S.B3 | Biolegend | 502506 | 1:30 |
| TNF-α | PE-Cyanine 7 | MAb11 | Thermofisher | 25-7349-82 | 1:65 |
| IL-2 | APC | MQ1-17H12 | Biolegend | 500310 | 1:30 |
| CD8a | PE | RPA-T8 | Thermofisher | 12-0088-42 | 1:30 |
